# Discovery of ravenelin B from *Exserohilum rostratum*: structural elucidation of a scarce xanthone via integrated NMR/DFT-GIAO approach and comprehensive excited-state characterization

**DOI:** 10.1007/s00894-026-06810-8

**Published:** 2026-07-03

**Authors:** Patrícia S. B. Marinho, Neidy S. S. dos Santos, Catharina B. de Araújo, Marcelo R. S. Siqueira, José E. S. Siqueira, Andersson Barison, Francinete R. Campos, Mayra Pinheiro, Rodrigo Gester, Andrey M. R. Marinho

**Affiliations:** 1 Programa de Pós-Graduação em Química, Universidade Federal do Pará, Rua Augusto Corrêa, 01-Guamá, Belém, 66075-110 Pará Brazil; 2 Programa de Pós-Graduação em Química, Universidade Federal do Sul e Sudeste do Pará, Marabá, 68507-590 Pará Brazil; 3 Instituto de Física, Universidade Federal de Alagoas, UFAL, 57072-970 Maceió, AL Brazil; 4 Instituto de Pesquisas Energéticas e Nucleares, IPEN/CNEN, 05508-000 São Paulo, SP Brazil; 5 Universidade Federal do Amapá, Departamento de Ciências Exatas e Tecnológicas, Macapá, 68903-419 Amapá Brazil; 6 Programa de Pós-Graduação em Química, Universidade Federal do Paraná, Curitiba, 81530-900 Paraná Brazil; 7 Departamento de Farmácia, Universidade Federal do Paraná, Curitiba, 80060-000 Paraná Brazil; 8 Faculdade de Física, Universidade Federal do Sul e Sudeste do Pará, Marabá, 68507-590 Pará Brazil

**Keywords:** Xanthones, *Exserohilum rostratum*, DFT/GIAO, NMR simulation, Scarce natural products, Endophytic fungi

## Abstract

**Context:**

This study reports the isolation and structural characterization of ravenelin (RVL) and ravenelin B (RVL B), a novel xanthone derivative produced by the endophytic fungus *Exserohilum rostratum* from the Brazilian Amazon. The compound was obtained in minute quantities with partial purity, presenting significant challenges for traditional structural characterization. Through the combined application of NMR spectroscopy (1D/2D) and DFT/GIAO calculations, we achieved unequivocal structural determination, demonstrating an effective strategy for investigating scarce natural products. Extensive computational analysis revealed key insights into the molecular properties: RVL B exhibits a predominantly locally excited character (61%) compared to RVL’s mixed charge-transfer/locally excited nature and shows promising two-photon absorption (TPA) properties with a high cross-section (351 GM in water) for higher excited states. Further TD-DFT calculations elucidated the solvent-dependent photophysical behavior, including absorption/emission spectra and Stokes shifts. These findings not only expand the chemical diversity of Amazonian endophytes but also establish a comprehensive framework for characterizing low-abundance metabolites using integrated experimental and theoretical approaches, while revealing potential applications as substituent design in nonlinear optics and photodynamic therapy.

**Methodology:**

This work discusses the procedure for the isolation, characterization, and theoretical insights into the electronic properties of a novel xanthone isolated from an Amazonian fungus. High-resolution mass spectra were obtained in positive ion mode using a MicroTOF-Q mass spectrometer (Bruker Daltonics, USA) equipped with an electrospray ionization (ESI) source. NMR spectra were recorded on a Bruker Ascend 400 using the solvent signal (*d*-chloroform) as a reference. Chemical shifts are given in delta (δ) values, and coupling constants (*J*) are given in Hertz (Hz). Concerning theoretical methods, all molecular optimizations and NMR calculations were performed within the DFT/GIAO framework using ωB97X-D/6-311++G(*d*, *p*). When necessary, the solvent was accounted for by the integral equation formalism of the polarizable continuum model. The electronic excitations of the ground and first excited state, as well as two-photon absorption cross-sections, were calculated within the TD-DFT framework by using different degrees of Hartree-Fock exchange (CAM-B3LYP, ωB97X-D, and M06-2H), also associated with the Pople 6-311++G(*d*, *p*) basis set and the continuum model of solvation.

**Supplementary Information:**

The online version contains supplementary material available at 10.1007/s00894-026-06810-8.

## Introduction

Filamentous fungi are a vital source of structurally diverse secondary metabolites with significant biological potential [1], among which xanthones stand out due to their broad occurrence and remarkable bioactivities. The pharmacological relevance of xanthones is well-documented, including antibacterial, cytotoxic, antifungal, and hepatoprotective properties, particularly against paracetamol-induced toxicity [2–4]. This is exemplified by chlorinated xanthones from *Penicillium citrinum* HL-5126, which showed potent activity against *Vibrio parahaemolyticus* [5], and derivatives like 2-hydroxy-6-formyl-vertixanthone that are effective against methicillin-resistant *Staphylococcus aureus* [2]. Further underscoring the class’s potential, well-known xanthones such as mangostin, gentisein, and garcinone E display antimicrobial, anticancer, and neuroprotective effects [6], highlighting that each newly discovered derivative may represent a promising pharmaceutical lead.

**Fig. 1:**
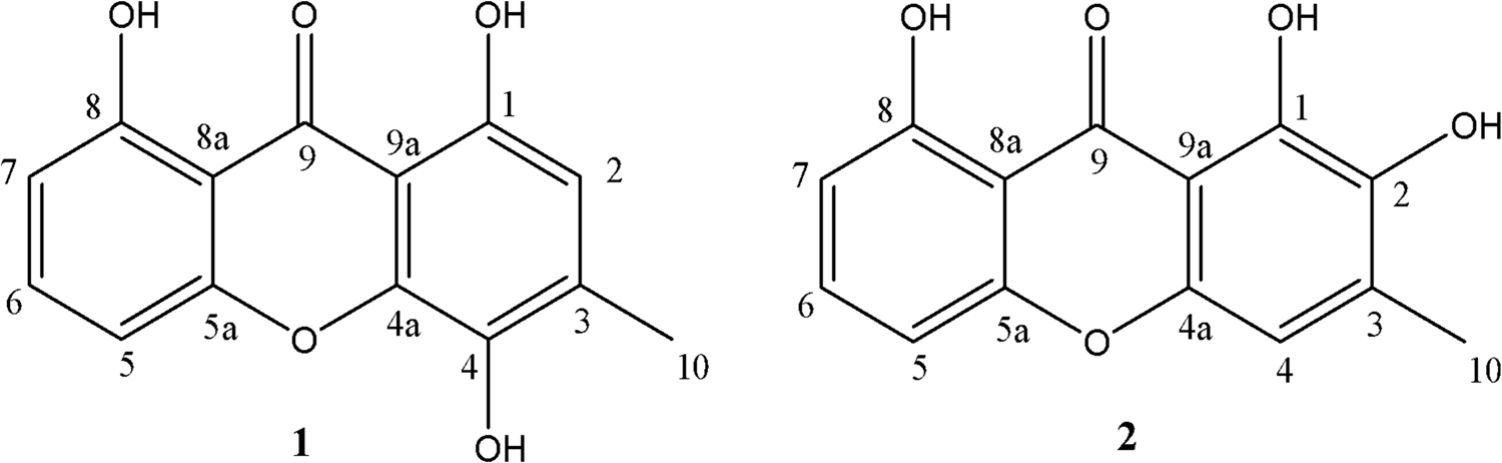
Ravenelin (RVL: 1,4,8-trihydroxy-3-methyl-9H-xanthen-9-one) and ravenelin B (RVL B: 1,2,8-trihydroxy-3-methyl-9H-xanthen-9-one)

However, despite their therapeutic promise, the structural complexity of many secondary metabolites, like xanthones, might be a significant challenge for characterization. Sometimes, it gets hard to interpret the intricate NMR spectra of these compounds for many reasons: low-purity samples and scarce mass make it hard to characterize and perform biological and physicochemical assays. To overcome this limitation, computational approaches have emerged as indispensable tools. By enabling the simulation of 1D and 2D NMR spectra, these methods now play an essential role in resolving the structures of organic molecules [7–14].

Besides biological finalities, xanthones and xanthene-like molecules have gained more attention due to their photophysical utilities. One of these properties is the Stokes shift, taken as the difference between the maxima of the emission and absorption spectra ($\Delta \lambda = \lambda _\text {em}-\lambda _\text {max}$). In such a context, α-mangostin, a xanthone-like molecule, stands out as a pronounced case since a significant Stokes shift (∼200 nm) has been reported not only for this compound but also for its derivatives [15]. These features have signaled strategic photodynamic uses such as biological probes and sensors, for example, [16, 17].

Two-photon absorption (TPA) is another optical property that has garnered attention in xanthones and xanthene molecules. Essentially, this is a nonlinear optical (NLO) process that occurs near the infrared wavelength and, therefore, causes low damage to organic tissues [18, 19]. Thus, molecules with higher TPA cross-sections ($\sigma ^{TPA}$) are often used not only in 3D optical data storage, photonics, and nonlinear optical devices but also in biological imaging and photodynamic therapy. Typically, cross-section values between 50 and 1000 GM are already considered interesting and the starting point for these applications.

While positional isomers of xanthones are known, the ortho vs. para relocation of hydroxyl groups can induce significant changes in photophysical properties due to differing HOMO-LUMO overlap and charge-transfer (CT) vs. locally excited (LE) character. Preliminary TD-DFT calculations reveal that RVL B exhibits LE character (61%) predominantly compared to RVL’s mixed CT nature (44% CT), resulting in Stokes shifts up to 400 nm in the gas phase vs. 100 nm for RVL, plus a superior two-photon absorption cross-section ($\delta _\text {TPA}$ up to 351 GM in water). These isomeric differences distinguish RVL B from previously reported xanthones, positioning it as a unique candidate for nonlinear optics and photodynamic probe applications.

With this framework in mind, our study focuses not on biological applications, but on the isolation and structural determination, reactivity, and the photophysics of ravenelin (RVL) and a novel xanthone, ravenelin B (RVL B), derived from *E. rostratum* biomass. To achieve this, we integrated spectroscopic and computational techniques to elucidate molecular structures even when sample quantities are limited or purity is suboptimal. Beyond elucidating the molecular structure, this work takes advantage of quantum chemical methods like density functional theory (DFT) to understand the photodynamics and NLO response of the compounds of interest. Notably, different levels of theory indicate a higher Stokes shift ($\Delta \lambda > 50$ nm) and, particularly for RVL B, an improved TPA cross-section ($\sigma ^{TPA} > 300$ GM) is estimated. These results suggest possible applications beyond traditional pharmacological uses.

## Methodology

### General procedures

High-resolution mass spectra were obtained in positive ion mode using a MicroTOF-Q mass spectrometer (Bruker Daltonics, USA) equipped with an electrospray ionization (ESI) source. NMR spectra were recorded on a Bruker Ascend 400 using the solvent signal (d-chloroform) as a reference. Chemical shifts are given in delta (δ) values, and coupling constants (*J*) are given in Hertz (Hz).

### Microorganism

The fungus *E. rostratum* was obtained from a collection of the “Laboratório de Bioensaios e Química de Microrganismos – LaBQuiM/UFPA.” One strain is deposited as code ER11. The fungus *E*.*rostratum* was identified by DNA sequencing at the Institute of Biological Sciences at UFPA [20].

### Culture of *E. rostratum* in rice and isolation

Twenty-five Erlenmeyer flasks (500 mL) containing 90 g of rice (“Uncle Ben’s”) and 75 mL of distilled water per flask were autoclaved at $121^\circ$C for 45 min. Small cubes of PDA medium containing *E*.*rostratum* mycelium were added to 12 Erlenmeyer flasks under sterile conditions. Three flasks served as controls. After 25 days of growth at 25 $^\circ$C, the biomass obtained was macerated with ethyl acetate. After filtration, the solution obtained was concentrated in a rotary evaporator to obtain the biomass extracts. The ethyl acetate extract (4.1 g) was fractionated on a silica gel chromatography column eluted with hexane, ethyl acetate, and methanol in a polarity gradient to obtain F1 (hexane), F2 (hexane/OEtAc 8:2), F3 (hexane/OEtAc 1:1), F4 (hexane/OEtAc 2:8), F5 (OEtAc), and F6 (MeOH). The compounds ravenelin (1) 15 mg and revenelin B (2) 01 mg were isolated from fraction F3 after successive fractionation on a silica gel column.

### Theoretical methodology

Geometry optimizations and vibrational calculations were performed in the gas phase and in solvent environments (CDCl$_3$ and water) using the integral equation formalism of the polarizable continuum model (IEF-PCM) [21] in conjunction with density functional theory and the 6-311++G(*d*, *p*) basis set. The absence of imaginary frequencies confirmed that the optimized geometries correspond to true local minima on the potential energy surface.

Validating the experimental NMR parameters was an essential step, especially for RVL B, which could only be isolated with low purity. Therefore, the theoretical analysis of the nuclear magnetic resonance (NMR) spectra was carried out within the Gauge-Invariant Atomic Orbital (GIAO) formalism [22]. Initially, we relied on the established performance of B3LYP geometries for describing $^1$H and $^{13}$C NMR parameters [23]. However, since xanthones are large molecules that exhibit intramolecular hydrogen bonding, we also assessed the performance of geometries obtained with ωB97X-D, which accounts for a different fraction of Hartree-Fock exchange and includes dispersion corrections.

Once the RVL and RVL B geometries were determined, the electronic absorption and fluorescence were subsequently investigated within the time-dependent formalism of density functional theory (TD-DFT) [24]. At this stage, all xanthone geometries were again optimized in the ground and first excited state, but now using DFT methods with a higher fraction of HF exchange (CAM-B3LYP, 65%; ωB97X-D, 97%; M06-HF, 100%), since this feature is essential to correct the classical underestimation of B3LYP for $\pi \pi ^*$ and CT states, correctly describing the differential stabilization of HOMO/LUMO in polar media [29, 30]. Given the agreement among the DFT methods cited above, the two-photon absorption cross-section was analyzed in the light of the CAM-B3LYP method, with special focus on the solvent contributions, again considering the IEF-PCM formalism.

Finally, the results obtained from the TD-DFT calculations were subsequently employed for the quantitative analysis of interfragment charge transfer (IFCT). In the present study, the hole and electron distributions were calculated using the IFCT method, employing the Hirshfeld partition in the Multiwfn 3.8 software [31–33]. All molecular optimization and electronic excitations were carried out using the Gaussian 16 program [34]. On the other hand, two-photon absorptions were estimated taking advantage of the Dalton program [35].

## Results

### Isolated compounds

Fractionation in CC of the ethyl acetate biomass extract of *E*.*rostratum* led to the isolation of the compounds ravenelin (RVL) and ravenelin B (RVL B) (Fig. 1). Compound RVLB was obtained in few amounts (01 mg). Moreover, to obtain more amount of RVLB, more extract was produced by recultivating the fungus, but it was not possible to obtain RVLB again. The structures of the isolated compounds were determined by 1D and 2D NMR, MS analysis, and computational simulation.

### Structural elucidation

#### Experimental NMR analysis

RVL was previously isolated by our group, and its antiprotozoal activity and NMR data were reported in earlier work [36]. In the present study, we isolated the xanthone RVLB, whose $^1$H and $^{13}$C NMR data closely resemble those of ravenelin (RVL).

**Fig. 2:**
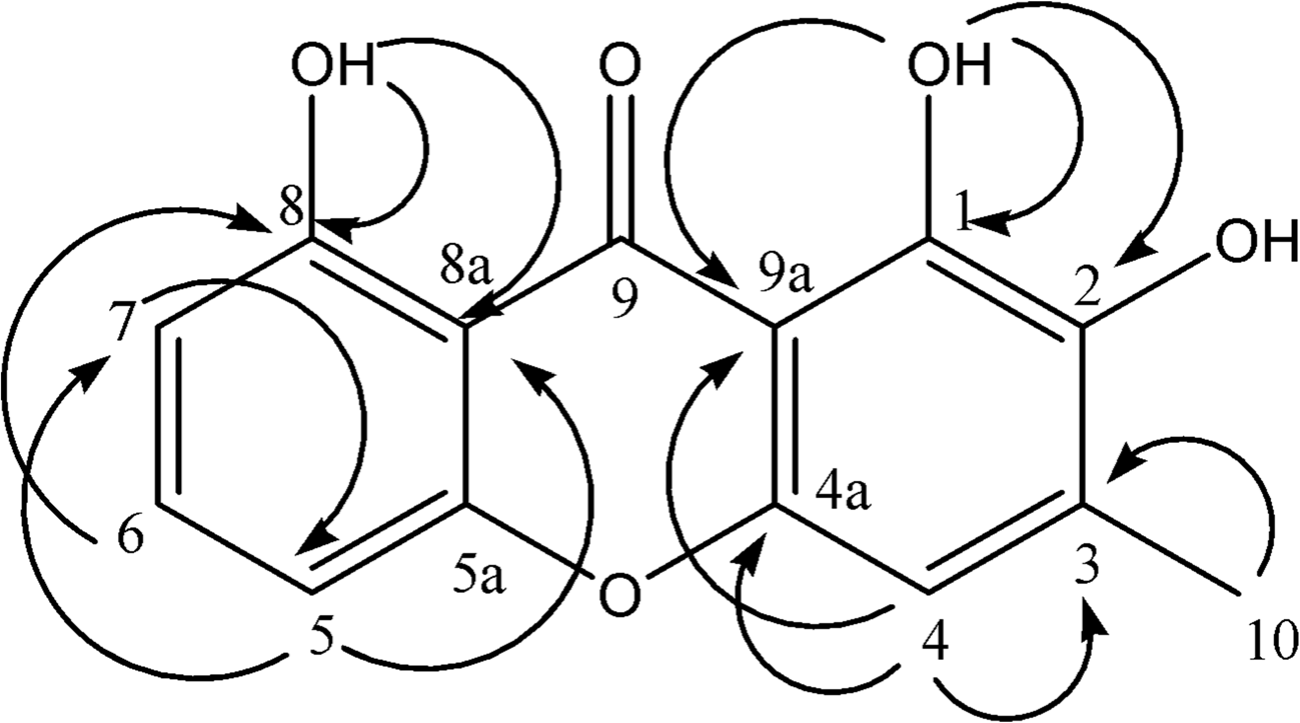
Heteronuclear multiple bond correlation (HMBC) observed for ravenelin B (RVL B)

RVL B was obtained as an orange solid, and its $^1$H NMR spectrum displayed characteristic aromatic signals. Notably, the signal at δ 7.59 (dd, *J* = 8.4, 8.3 Hz), assigned to H-6, is ortho-coupled to H-5 (δ 6.90, dd, *J* = 8.4, 0.9 Hz) and H-7 (δ 6.77, dd, *J* = 8.3, 0.9 Hz). The meta-coupling between H-5 and H-7 (*J* = 0.9 Hz) further supports the presence of a 1,2,3-trisubstituted aromatic ring in this moiety.

A second aromatic system was identified by the signal at δ 6.79 (q, *J* = 0.7 Hz), attributed to H-4, which exhibits long-range coupling with methyl Me-10 (δ 2.42) in the heteronuclear multiple bond correlation (HMBC) spectrum. Additionally, two singlet signals at δ 11.66 and δ 11.86 were assigned to the chelated hydroxyl groups OH-1 and OH-8, respectively, hydrogen-bonded to carbonyl C-9. Key HMBC correlations for OH-1 (δ 11.66) with carbons at δ 144.9 (C-1), δ 106.2 (C-9a), and δ 137.8 (C-2) reinforced this assignment. Similarly, Me-10 showed HMBC correlations with δ 135.4 (C-3), δ 108.1 (C-4), and δ 137.8 (C-2). Together, these observations unambiguously position the non-chelated hydroxyl OH-2 at C-2 (Fig. 2). Despite the low signal-to-noise ratio in the $^{13}$C NMR spectrum, the carbonyl carbon C-9 was confidently assigned to δ 182.5.

**Table 1:** H and $^{13}$C NMR data for compounds RVL and RVLB (400 MHz, CDCl$_3$) and theoretical chemical shifts using ωB97X-D/6-311++G(d,p), with simulations in chloroform (IEF-PCM) and GIAO. Chemical shifts are relative to TMS

	RVL exp.		RVL B exp.		RVL theo.		RVL B theo.	
Nuclei	$^1$H (mult, J)	$^{13}$C	$^1$H (mult, J)	$^{13}$C	$^1$H	$^{13}$C	$^1$H	$^{13}$C
1		151.7*		144.9		153.91		155.6
2	6.64 (s)	112.2		137.8	6.89	110.07		145.9
3		136.9*		135.4		137.91		136.4
4		136.2	6.79 (q, 0.7)	108.1		132.94	7.00	110.1
4a		138.8		148.9		139.61		154.2
5	7.01 (d, 8.4)	108.2	6.90 (dd, 8.4, 0.9)	107.2	7.07	104.17	7.04	113.4
5a		153.7		156.4		155.52		164.8
6	7.72 (t, 8.3, 8.2)	137.5	7.59 (dd, 8.4, 8.3)	137.2	7.93	139.29	7.94	140.0
7	6.78 (d, 8.0)	111.4	6.77 (dd, 8.3, 0.9)	110.2	7.05	109.32	7.00	113.2
8		157.1		161.3		163.18		165.2
8a		108.4		107.6		103.76		113.8
9		187.1		182.5		186.18		187.4
9a		106.9		106.2		101.88		112.2
10	2.37 (s)	17.1	2.42 (q, 0.7)	16.7	2.05; 2.54	13.14	2.05; 2.53	15.7
OH-1	10.96 (s)		11.66 (s)		10.77		11.42	
OH-8	8.45 (sl)		11.86 (s)		11.78		11.69	

Legend: *H*, hydrogen; *C*, carbon; δ, chemical shift (ppm). TMS shielding (H): 31.8821; TMS shielding (C): 182.4656

Carbonyl groups typically have a longer relaxation time in $^{13}$C NMR, and therefore, their signals tend to be less intense than other types of $^{13}$C in molecules. In the spectrum, even with low intensity, we observed the beginning of a signal with a chemical shift typical of a carbonyl group, and therefore, we attributed this value to C9. Due to the small sample size, even repeating the experiment with a greater number of pulses, it was not possible to improve the spectrum. To overcome this limitation, we applied apodization, which is a mathematical filter applied to raw NMR data (Free Induction Decay, FID) to suppress background electronic noise while preserving the real signal (see **Supplementary Material**). In practice, the original data are multiplied by a decaying function that retains the beginning of the acquisition (where the molecular signal is strongest) and progressively attenuates the end (where only instrument noise remains). For carbons with long relaxation times, such as C-9, the emitted signal is weak and prolonged, easily hidden by baseline noise. By applying exponential multiplication with increasing line broadening (LB) parameters (e.g., LB = 1.0 → 15.0 Hz), we mathematically silenced the noise and revealed a consistent, well-defined signal centered at 182.5 ppm. This unequivocally demonstrates that the baseline elevation at that position corresponds to a real physical signal, not random noise or an artifact, thus validating the structural assignment.

The proposed molecular formula C$_{14}$H$_{10}$O$_{5}$ for RVL B was confirmed by HR-ESIMS (+) (m/z 259.0619 [M+H]$^+$) and NMR data. Comparison with literature values for ravenelin [36, 37] revealed distinct chemical shifts for C-1, C-2, and C-3, supporting the presence of a non-chelated hydroxyl at C-2 in 2, contrasting with ravenelin’s C-4 substitution. Given the limited sample quantity and resulting poor NMR signal quality, we further validated the structure through simulated $^1$H and $^{13}$C NMR spectra. Based on these analyses, 2 was identified as a novel natural product and designated ravenelin B. Full $^1$H and $^{13}$C NMR data are provided in Table 1.

#### 1,2,8-Trihydroxy-3-methylxanthone—ravenelin B (RVL B)

NMR data (400 MHz, CDCl3): see Table 1; HR-ESIMS (full scan, positive ion mode) m/z: found 259.0619 [M+H]+; Calc. for C14H10O5: 259.0606 [M+H]$^+$.

### DFT verification

#### Computational NMR analysis

An accurate understanding of the experiment depends on how closely the theoretical geometry reflects reality. After analyzing the root-mean-square deviation (RMSD) between theoretical and experimental data for different DFT methods (see Fig. 6), we decided in favor of the ωB97X-D results, as this method showed the best coefficient of determination ($R^2$) values for both $^1$H and $^{13}$C in RVL and RVL B, thus validating their structures. Therefore, although B3LYP results were calculated and are shown in the Supplementary Material, all geometric parameters are discussed for ωB97X-D geometries.

**Fig. 3:**
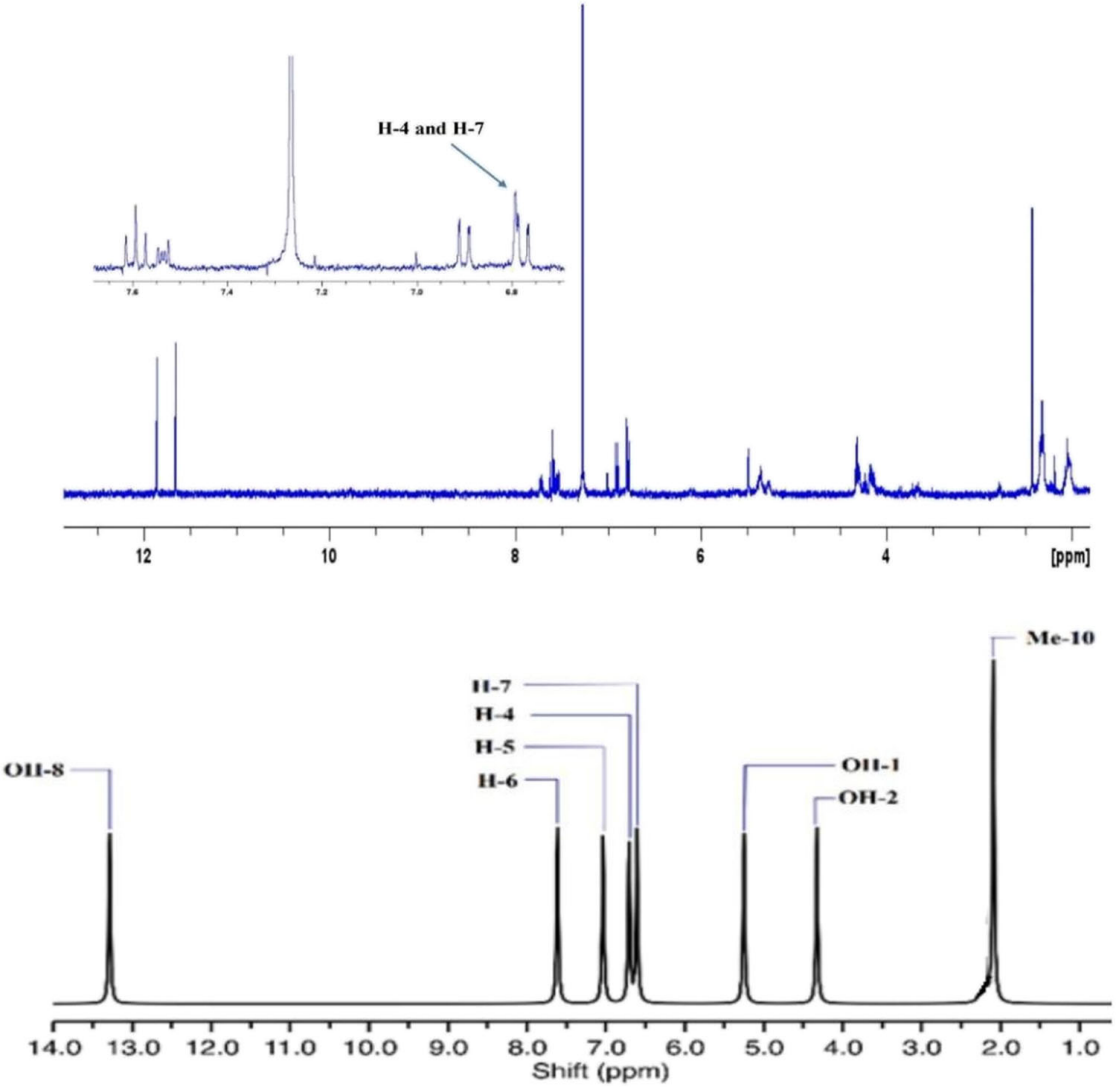
Comparison between the experimental (blue) and theoretical (black) $^1$H NMR spectra of RVL B. There is a similarity between the spectra, which corroborates the proposed structure for RVL B

**Fig. 4:**
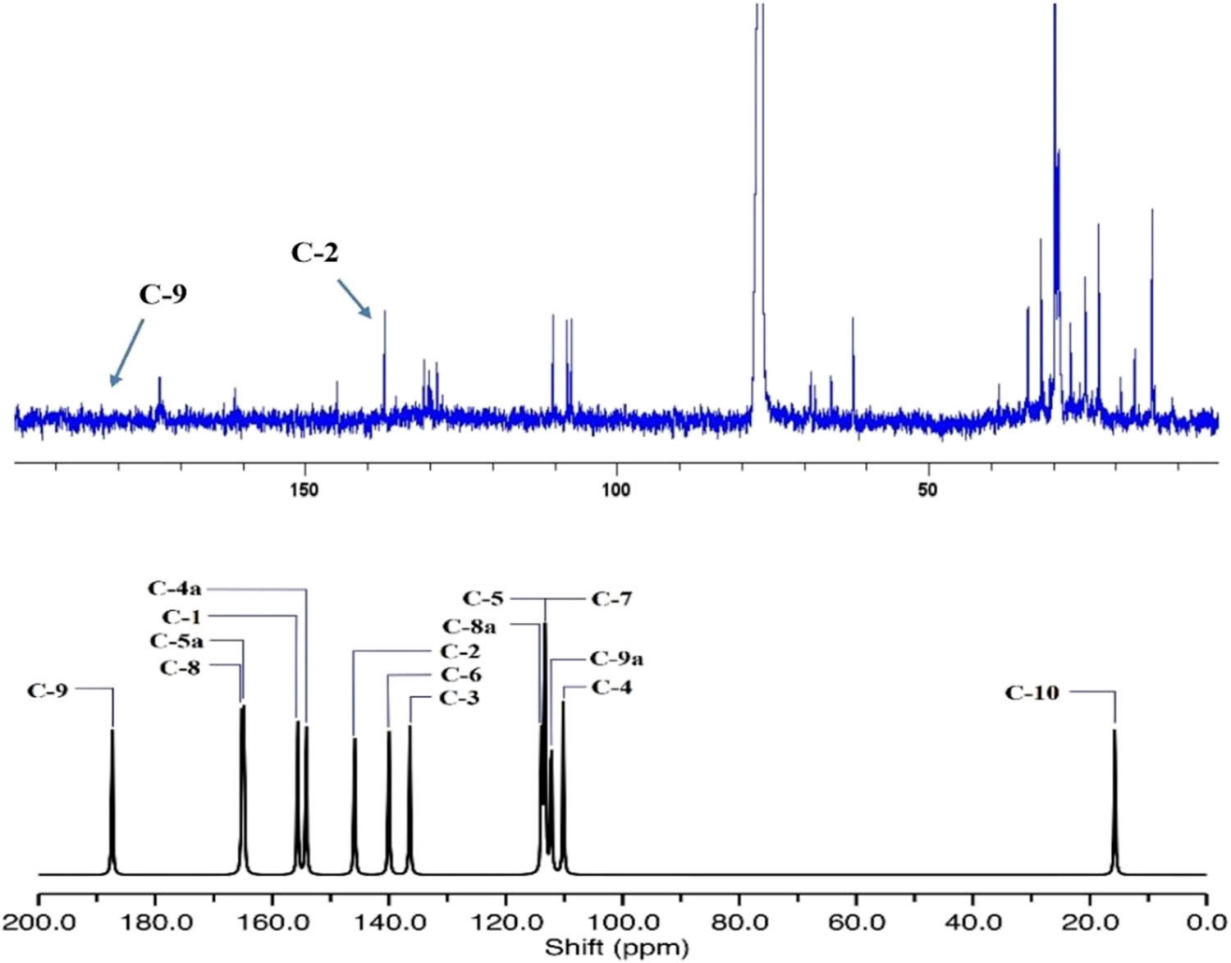
Comparison between the experimental and theoretical $^{13}$C NMR spectra RVL B. There is a similarity between the spectra, which corroborates the proposed structure for RVL B

RVL B was obtained in small amounts and with low purity, 10%, resulting in NMR spectra with many interfering signals, so that for correct assignment of the $^1$H and $^{13}$C NMR signals, it was necessary to use computational methods to simulate the NMR spectra for compounds RVL and RVL B. For both compounds, agreement between theoretical and experimental data was observed. RVL is already known, and its NMR data are correctly attributed in the literature, so it was used as a standard to obtain the best theoretical parameters to calculate the spectra of RVL B. The theoretical NMR data for RVL B showed an average rate of change of chemical shift values of $\Delta = \pm 4\%$, a variation that can be considered low and shows the viability of the method used [4], which helped to confirm the proposed structure and the correct assignment of the NMR signals for RVL B. Figures 3 and 4 show the experimental and theoretical $^1$H and $^{13}$C NMR spectra forRVL B.

**Fig. 5:**
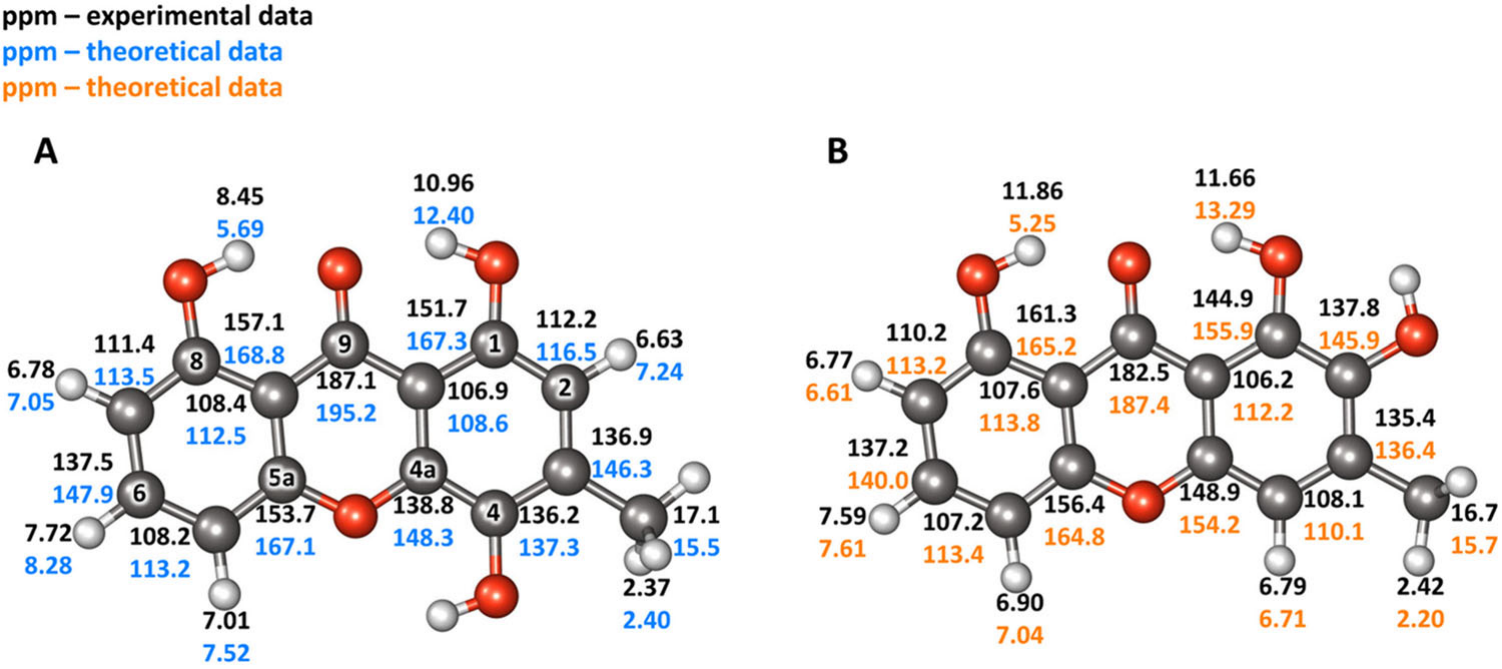
Comparison of experimental and theoretical $^1$H and $^{13}$C NMR data for ravenelin (RVL) and ravenelin B (RVL B)

As can be seen in the experimental $^1$H NMR spectrum of RVL B, the signals for the hydrogens H-4 and H-7 are superimposed, similar to what was observed in the simulated spectrum, where it can be seen that the signals for these hydrogens have chemical shifts very close to each other (Fig. 3). The simulated $^{13}$C NMR spectrum of RVL B (Fig. 4) shows a good approximation to the experimental spectrum, where the deshielding effect caused by the OH group in C-2 can be seen in the theoretical spectrum when compared to the C-2 signals of RVL (Table 1, Fig. 4).

**Fig. 6:**
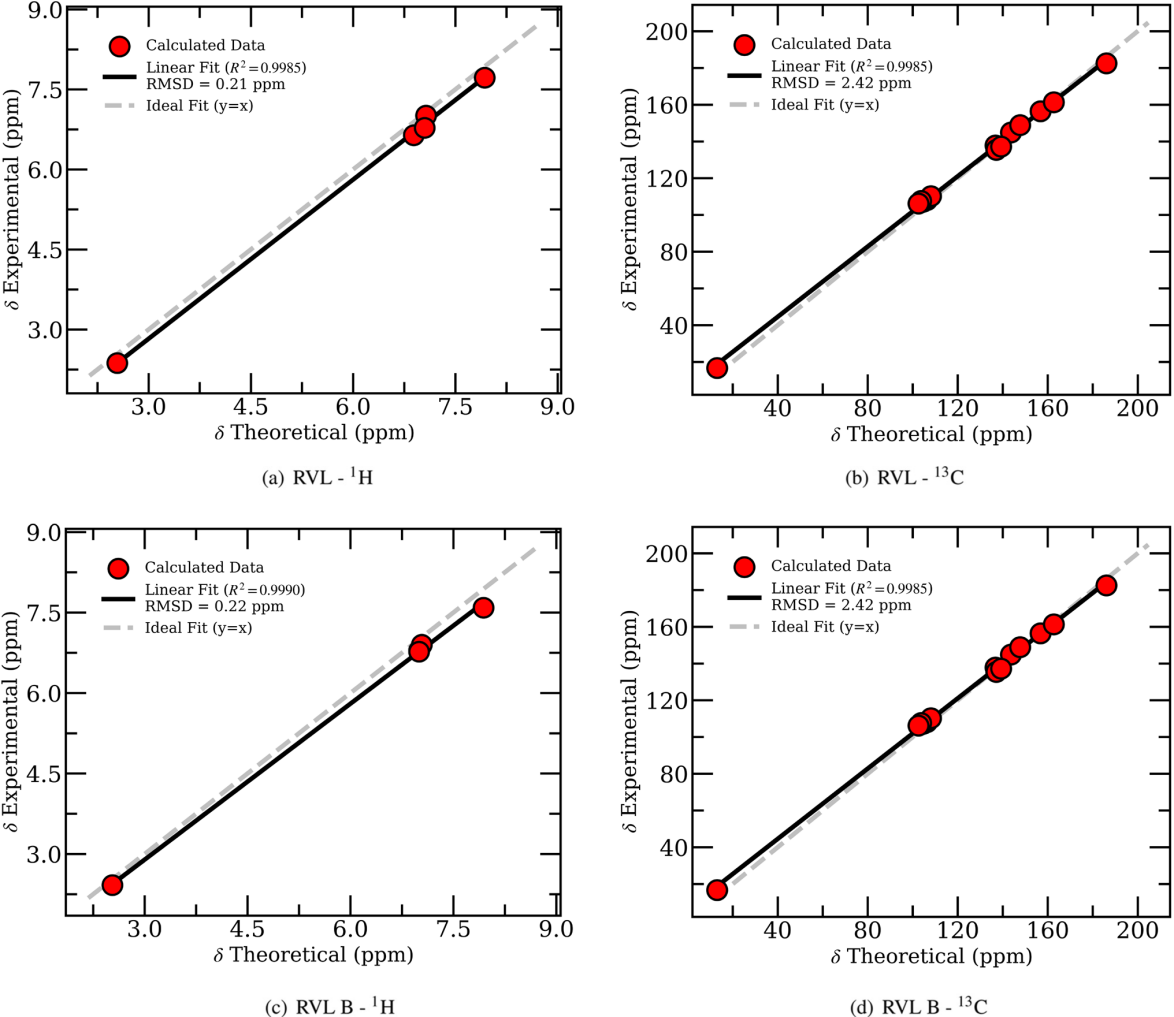
Linear correlations between experimental and theoretical data for $^{1}$H and $^{13}$C chemical shifts in RVL and RVL B. Theoretical calculations were performed at the ωB97X-D/6-311++G(d,p) level using the GIAO approach and the IEF-PCM solvation model (chloroform). RMSD values quantify the agreement between datasets, where lower values indicate better predictive performance. Chemical shifts (δ) are reported relative to tetramethylsilane (TMS) using the calculated shielding constants: $\sigma _{ref}$ (H) = 31.8821 and $\sigma _{ref}$ (C) = 182.4656

Figure 5 shows the chemical shifts (δ) obtained for the xanthones studied; as can be seen, there are some small discrepancies in the magnitude of δ for some chemical elements. This occurs when some relevant effects are not taken into account in the quantum chemical calculations. These effects include long-range and dispersion corrections, as well as explicit contributions from the solvent, such as solute relaxation and polarization due to the solvent. It has been discussed that these interactions can improve both shielding constants and spin-spin coupling parameters [38, 39]. However, the current DFT discussion allows a good understanding of the experimental spectra. For example, Fig. 5 points out the linear regression lines for compounds RVL and RVL B. The corresponding angular coefficients, 1.041 ($\tan ^{-1}146.1^{\circ }$) and 1.095 ($\tan ^{-1}147.6^{\circ }$), indicate angles close to 45$^{\circ }$. This finding suggests that the system approximates an equilateral triangle configuration. Thus, the theoretical model agrees well with the experimental results (Fig. 6).

#### Computational geometry analysis

**Fig. 7:**
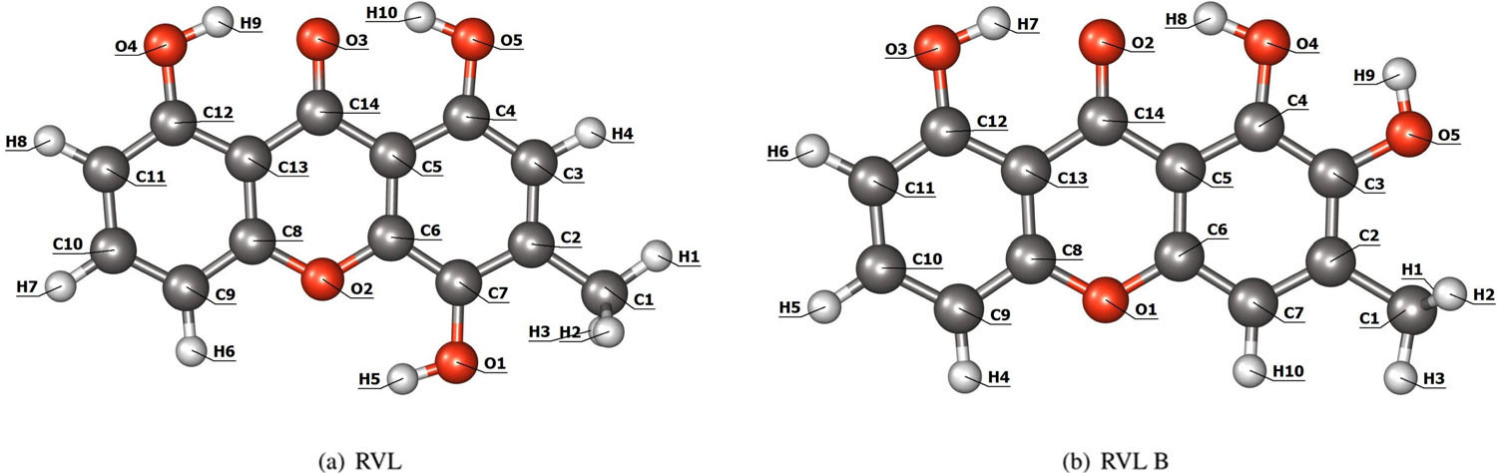
Atom numbering used for the analysis of bond lengths and bond length alternation (BLA)

Molecules with electron donor and acceptor groups connected by a π-bridge are susceptible to structural and electronic changes in polar environments[40, 41]. Variations in bond lengths influence the bond length alternation (BLA), where high values indicate stronger localization of π-electrons, while low values reflect increased delocalization and resonance. Table 2 and Fig. 7 present the bond lengths of compounds 1 (RVL) and 2 (RVL B), along with their BLA coordinates, which monitor the structural characteristics of the conjugated rings and hydroxyl groups.

**Fig. 8:**
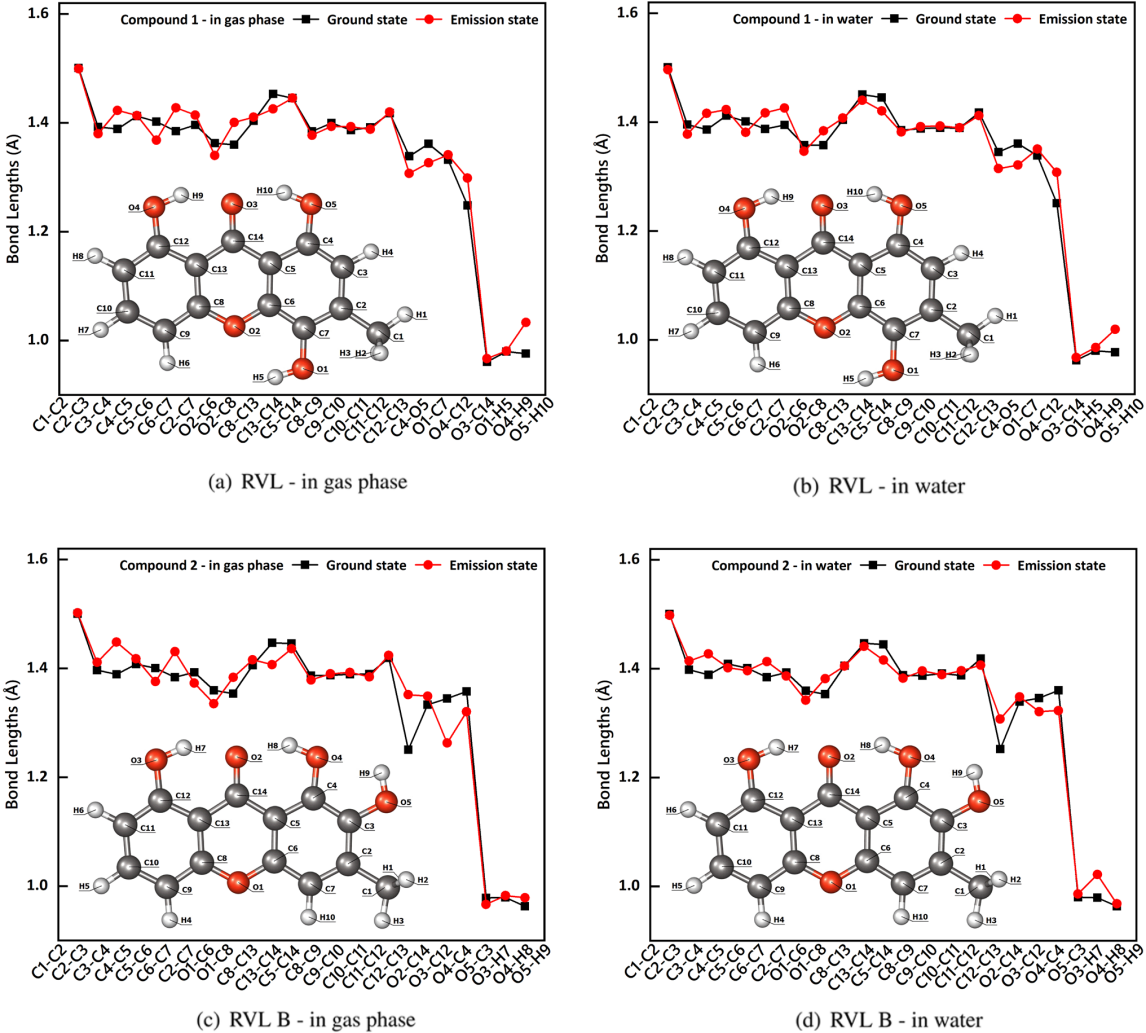
Bond lengths of the compounds in the ground and emission states, calculated in the gas phase and in water using the ωB97X-D/6-311++G(d,p) level of theory. This highlights the differences between the two states in each environment

The solvation (gas phase to water), minimal changes in bond lengths are observed for both molecules. In RVL B (gas phase), slight elongations are seen for C2–C3 ($+0.00425 {\AA }$) and C3–C4 ($+0.00137 {\AA }$), while C1–C2 ($-0.00043 {\AA }$) and C4–C5 ($-0.00546 {\AA }$) exhibit small contractions compared to RVL (Table 2 and Fig. 7). The O2–C6 ($-0.00277 {\AA }$) and O2–C8 ($-0.00668 {\AA }$) bonds also shorten, possibly due to intramolecular electronic effects and resonance stabilization. Both systems display only minor geometric variations when moving from the gas phase to aqueous solution (Fig. 8).

The most noticeable change in RVL occurs in χA1, which shifts from $-0.00184 {\AA }$ (gas phase) to $-0.00341 {\AA }$ Å (water), indicating a subtle enhancement in π-delocalization along the central ring system (Table 2). This suggests that solvation slightly perturbs the electronic distribution even though the geometrical rearrangements remain small. The rigidity of the fused ring framework helps maintain structural stability, resulting in minimal geometric distortion upon environmental change. Therefore, the structural differences primarily arise from electronic polarization effects rather than from large-scale geometry modifications.

### Electronic excitation analysis

#### Ground state absorption spectra

**Fig. 9:**
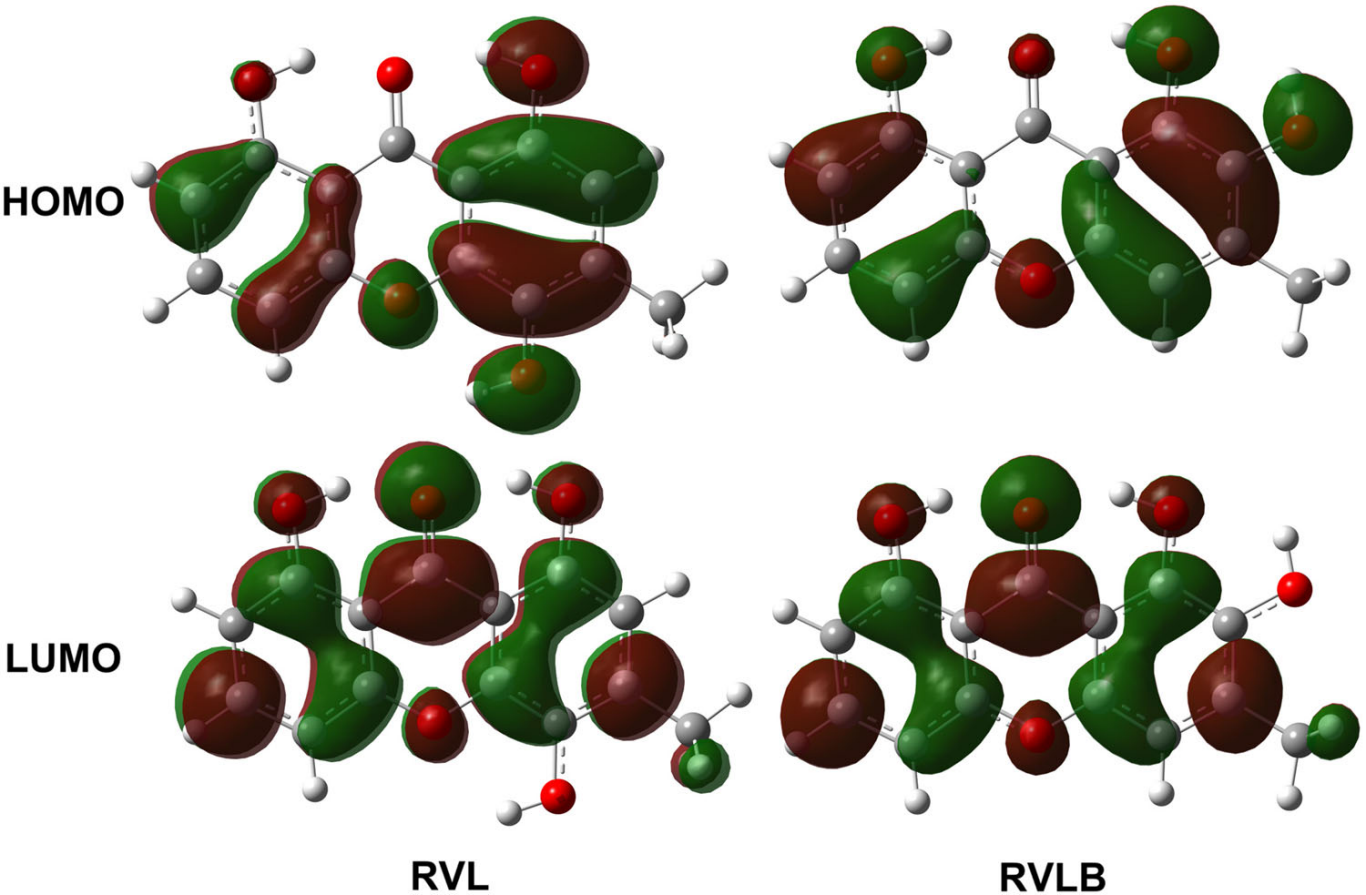
The compounds frontier molecular orbitals (in *e*V) at the ωB97X-D/6-311++G(*d,p*) level of gas phase

Table 3 shows the calculated results for the lowest $\pi \rightarrow \pi ^*$ electronic absorption. According to the prediction of CAM-B3LYP for ravenelin A at vacuum, this excitation occurs at 346.54 nm with a moderate oscillator force (0.08). This intensity is not usual if one takes into account that the molecular orbital analysis indicates that this transition (H→L) has a $\pi \rightarrow \pi ^*$ symmetry (see Fig. 9).

The calculated values for the lowest electronic absorptions of RVL and RVL B (∼300–350 nm in polar solvents, with oscillator strengths f≈0.08-0.19) corroborate absorption spectra reported for aromatic xanthones in the literature, where long-wavelength maxima are around 335 nm in the solid phase and bands from 227 to 370 nm in solution, attributed to $^1(n,\pi ^{*})$ and $^1(\pi ,\pi ^{*})$ transitions sensitive to the medium [43]. The relocation of the OH group to C-2 in RVL B induces a minimal isomeric blue shift (∼3–4 nm), reflecting greater HOMO-LUMO overlap (Fig. 9) and dominant HOMO-LUMO character of $^1B_2(\pi ,\pi ^{*})$ symmetry, typical of hydroxylated derivatives. The modest red shift (∼8 nm) with increasing solvent polarity aligns with dielectric effects in rigid conjugated systems, confirmed by low bond length alternation (BLA ≈ 0.001–0.004, Table 2), suggesting geometric stability for nonlinear optics applications. These findings validate the experimental absorption spectra of isolated RVL B, positioning it as an efficient UV–Vis chromophore.

Normally, this excitation is intense and assigns a broad absorption band. Thus, in compromising with the accuracy of the results, we were forced to apply other DFT methods to confirm the position line, intensity, and symmetries of this transition. For example, the ωB97X-D and M06-2X approximations indicate absorptions, respectively, at 343.42 nm and 342.65 nm.

For ravenelin B, the relocation of one hydroxyl group generates a mild blue shift for the energy of this electronic absorption band. While CAM-B3LYP predicts an electronic excitation at 342.03 nm for the gas phase system, with ωB97X-D and M06-2H do 338.89 nm and 338.62 nm, respectively. Again, all methods indicate an H→L with an oscillator force around 0.07 and symmetry $\pi \rightarrow \pi ^*$. It means mild isomeric effects have mild impacts on the intensity of these transitions.

**Table 2:** Bond lengths and bond length alternation (BLA) coordinates of RVL and RVL B in the ground and emission states, calculated in the gas phase and in water using the ωB97X-D/6-311++G(*d,p*) level of theory

RVL	Gas phase		Water		RVL B	Gas phase		Water	
Bond	Ground state	Emission state	Ground state	Emission state	Bond	Ground state	Emission state	Ground state	Emission state
C1-C2	1.50066	1.49917	1.50070	1.49661	C1-C2	1.50037	1.50205	1.50041	1.49792
C2-C3	1.39239	1.37974	1.39544	1.37783	C2-C3	1.39668	1.41121	1.39794	1.41395
C3-C4	1.38876	1.42276	1.38612	1.41635	C3-C4	1.38901	1.44823	1.38853	1.42706
C4-C5	1.41216	1.41346	1.41187	1.42291	C4-C5	1.40784	1.41798	1.40870	1.40161
C5-C6	1.40209	1.36817	1.40146	1.38095	C5-C6	1.40066	1.37581	1.40072	1.39639
C6-C7	1.38471	1.42767	1.38738	1.41709	C6-C7	1.38369	1.43072	1.38401	1.41291
C2-C7	1.39596	1.41421	1.39479	1.42601	C2-C7	1.39284	1.37292	1.39275	1.38625
O2-C6	1.36243	1.34013	1.35779	1.34637	O1-C6	1.35970	1.33519	1.35933	1.34187
O2-C8	1.35983	1.40086	1.35774	1.38380	O1-C8	1.35347	1.38353	1.35309	1.38173
C8-C13	1.40359	1.41033	1.40402	1.40750	C8-C13	1.40573	1.41587	1.40523	1.40482
C13-C14	1.45302	1.42542	1.45057	1.44031	C13-C14	1.44704	1.40688	1.44662	1.44122
C5-C14	1.44549	1.44544	1.44533	1.42081	C5-C14	1.44565	1.43596	1.44453	1.41561
C8-C9	1.38409	1.37724	1.38500	1.38178	C8-C9	1.38676	1.37878	1.38768	1.38227
C9-C10	1.39949	1.39341	1.38842	1.39178	C9-C10	1.38751	1.39003	1.38698	1.39581
C10-C11	1.38668	1.39276	1.38967	1.39297	C10-C11	1.38895	1.39266	1.39125	1.38879
C11-C12	1.39167	1.38792	1.38905	1.38962	C11-C12	1.38937	1.38440	1.38753	1.39601
C12-C13	1.41768	1.41997	1.41740	1.41216	C12-C13	1.41904	1.42394	1.41826	1.40654
C4-O5	1.33870	1.30705	1.34524	1.31457	O2-C14	1.25054	1.35158	1.25228	1.30754
O1-C7	1.36128	1.32644	1.36060	1.32117	O3-C12	1.33342	1.34896	1.33918	1.34822
O4-C12	1.33202	1.34117	1.33852	1.35045	O4-C4	1.34462	1.26290	1.34582	1.32082
O3-C14	1.24817	1.29840	1.25078	1.30765	O5-C3	1.35754	1.32028	1.35999	1.32295
O1-H5	0.96073	0.96679	0.96246	0.96736	O3-H7	0.97847	0.96634	0.97983	0.98598
O4-H9	0.97945	0.98077	0.98006	0.98599	O4-H8	0.97923	0.98249	0.97913	1.02157
O5-H10	0.97572	1.03318	0.97698	1.01911	O5-H9	0.96275	0.97871	0.96342	0.96785
Bond length alternation					Bond length alternation				
χA1	−0.00184	0.04557	−0.00341	0.04120	χA1	−0.00532	0.02395	−0.00583	0.00179
χA2	0.00235	0.01371	0.00012	0.00360	χA2	−0.00173	−0.00545	−0.00208	−0.00550
χA3	0.00310	−0.00691	−0.00188	−0.00640	χA3	−0.00167	−0.00998	−0.00313	−0.00165
χO1	0.40055	0.35966	0.39814	0.35381	χO3	0.35495	0.38263	0.35935	0.36224
χO4	0.35257	0.36039	0.35846	0.36446	χO4	0.36540	0.28041	0.36669	0.29925
χO5	0.36299	0.27387	0.36826	0.29546	χO5	0.39479	0.34157	0.39657	0.35510

*The parameters for bond length alternation (BLA) are detailed in the **Supplementary Material, Definition** S1 and S2

We pay some attention to the solvent effects, particularly for the aqueous environment. The results shown in Table 3 indicate that the environment has mild impacts on the electronic absorption spectra. In fact, only a slight red shift (∼8 nm) is observed for both molecules in any of the solvents used. However, the intensity of these excitations grows around 0.2 according to all DFT methods.

#### Emission spectra and Stokes shifts

We investigated the fluorescence spectra of the corresponding xanthone molecules in the $S_0\rightarrow S_1$ state. Understanding the behavior of a chromophore in its excited states is fundamental to understanding the so-called Stokes shift, which is the difference between maxima of absorption and emission ($\Delta \lambda = \lambda _{em}-\lambda _{abs}$). Molecules with higher Stokes shifts ($\Delta \lambda > 50$ nm) are particularly important in photonics and optoelectronics, being used as sensors, dye markers, solar cells, and other applications [42]. Table 3 shows the DFT results collected for RVL and RVL B.

**Table 3:** The absorption and emission (λ/nm), energy (*E*/*e*V), and the oscillator strength (*f*) to RVL and RVL B, in the gas phase and water environment obtained using the DFT functional and the 6-311++G(*d*, *p*) basis set with the solvent effect described by the IEF-PCM

		Absorption ($\pi \rightarrow \pi ^*$)				Emission ($\pi \leftarrow \pi ^*$)				Stokes
Method	Medium	$\lambda _{abs}$ (nm)	*f*	*E*	Cont	$\lambda _{emi}$ (nm)	*f*	*E*	Cont	$\Delta \lambda$ (nm)
RVL										
CAM-B3LYP	Gas	346.54	0.08	3.58	H→L	452.47	0.06	2.74	H→L	105.93
	Water	347.36	0.10	3.57	H→L	436.64	0.17	2.84	H→L	89.28
ωB97X-D	Gas	343.42	0.09	3.61	H→L	430.01	0.07	2.88	H→L	86.59
	Water	344.51	0.11	3.53	H→L	424.82	0.18	2.92	H→L	80.31
M06-HF	Gas	297.36	0.13	4.17	H→L	391.75	0.17	3.16	H→L	94.39
	Water	298.32	0.16	4.16	H→L	408.47	0.34	45750	H→L	110.15
RVL B										
CAM-B3LYP	Gas	342.03	0.07	3.62	H→L	746.45	0.02	1.66	H→L	404.42
	Water	343.48	0.10	3.61	H→L	423.03	0.11	2.93	H→L	79.55
ωB97X-D	Gas	338.89	0.08	3.66	H→L	713.22	0.02	1.74	H→L	373.33
	Water	340.66	0.10	3.64	H→L	407.77	0.13	45750	H→L	67.11
M06-HF	Gas	296.06	0.12	4.19	H→L	562.42	0.07	2.20	H→L	266.36
	Water	297.82	0.16	4.16	H→L	389.04	0.19	3.19	H→L	91.22

Taking the ground state as reference, the systems suffer a strong displacement into the lower spectral region, but preserve their transition intensities. Considering firstly the RVL molecule in the gas phase, CAM-B3LYP predicts a $\pi \leftarrow \pi ^*$ emission at 452.47 nm, which arises with an intensity of 0.06. Clearly, this means a strong Stokes shift of 105.93 nm. On the other hand, the ωB97X-D approximation indicates that this emission should occur at a higher energy (430.01 nm), reducing the Stokes shift to 86.59 nm, which is still a large value. We also applied the M06-HF, that is, we accounted for 100% of Hartree-Fock exchange. In this case, we obtained an emission at 391.75 nm and $\Delta \lambda$ displacement of 94.39 nm.

**Fig. 10:**
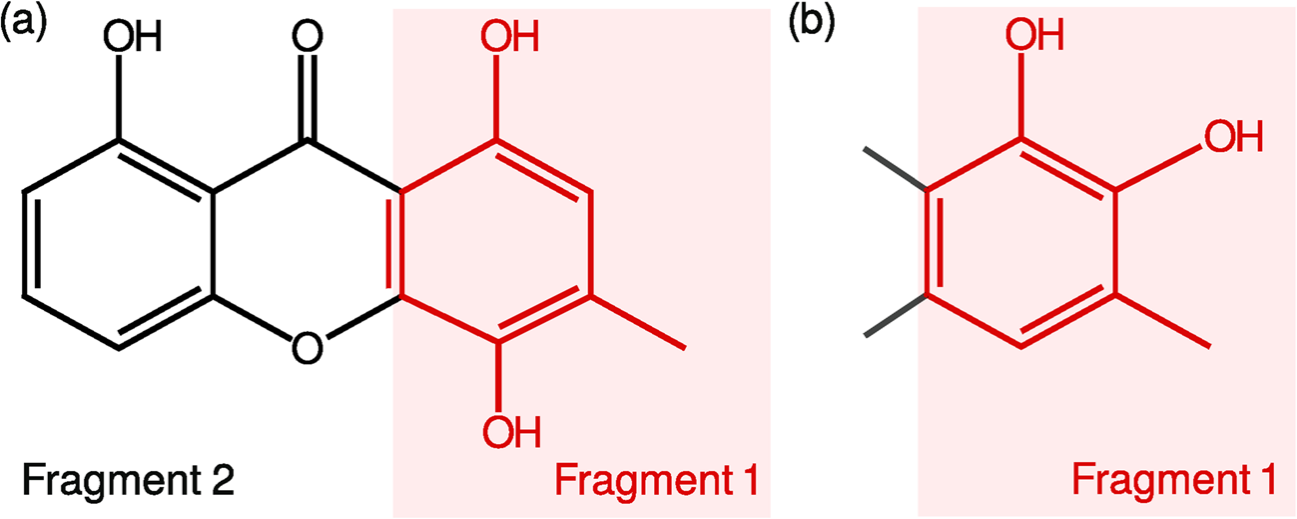
Decomposition of the isomers into two fragments for performing IFCT calculations: **a** fragment 1 corresponds to the 2-methylbenzene-1,4-diol group for RVL isomer, and **b** fragment 1 corresponds to the 3-methylbenzene-1,2-diol group for RVL B isomer. Fragment 2 comprising the C$_{7}$H$_{6}$O$_{3}$ part

The solvent effects were also analyzed. However, these results are not so conclusive for RVL, since CAM-B3LYP and ωB97X-D predict a slight reduction in the Stokes shift, while M06-HF signals a small increase. Possibly, the inclusion of explicit solute-solvent interaction would fix this problem. However, all methods indicate a Stokes shift ranging from 80.31 nm to 110.15 nm, which means mild solvent contributions.

On the other hand, RVL B seems more interesting, at least for lower dielectric constants. At vacuum conditions, one obtains values of 746.45 nm (CAM-B3LYP), 713.22 nm (ωB97X-D), and 562.42 nm (M06-HF) for the lowest $\pi \leftarrow \pi ^*$ emission. This means notable Stokes shifts between 266.36 and 404.42 nm. Unfortunately, this changes if the dielectric constant is increased to that corresponding to the water solvent. In such a case, $\Delta \lambda$ decreases considerably, assuming values that vary from 67.11 up to 91.22 nm.

The predicted Stokes shifts for RVL and RVL B (∼67-110 nm in water, up to 106–404 nm in gas phase) are consistent with emissive properties of aromatic xanthones, where fluorescence and phosphorescence originate from triplet states $^3(n,\pi ^{*})$ in non-polar media and $^3(\pi ,\pi ^{*})$ in polar ones, with moderate emissive intensities (f≈0.1-0.3) [43]. RVL B stands out for elevated shifts in vacuum, contrasting with α-mangostin (∼200 nm in derivatives) and aligning with emissions ∼340–450 nm reported for porphyrin-xanthone dyads and xanthone derivatives [43, 44]. The observed solvent dependence, with reduced shifts in polar media, reflects the typical $n\pi ^{*} \rightarrow \pi \pi ^{*}$ state transition in aromatic ketones, validating the experimental fluorescence data obtained for scarce isolated RVL B.

These photophysical profiles reinforce RVL B’s potential as an efficient UV–Vis fluorescent probe, with structural stability confirmed by low bond length alternation (BLA, Table 2). The combination of solvent-dependent emissive properties, moderate oscillator strengths, and high two-photon absorption cross-section (TPA up to 351 GM in water for S$_4$) suggests promising applications as photodynamic probes and in photodynamic therapy, corroborating the experimental data for this scarce natural compound.

#### Locally excited and charge-transfer excited state

The TD-DFT results using ωB97X-D functional were further utilized in the analysis of IFCT during $S_0\rightarrow S_1$ transition. The hole and electron distributions were calculated by employing the Hirshfeld partition. Figure 10 illustrates how the molecules were fragmented for the IFCT calculations. The interfragment electron transfer was studied by dividing the entire molecule into two fragments, with fragment 1 representing the substituted benzene moiety (2-methylbenzene-1,4-diol in RVL and 3-methylbenzene-1,2-diol in RVL B) and fragment 2 comprising the remaining xanthone scaffold, 1-(2-hydroxy-6-methoxyphenyl)-1-ethanone, C$_{7}$H$_{6}$O$_{3}$ fragment. The net transferred electrons between fragments, for $S_0\rightarrow S_1$ transitions, are also presented (Table 4). RVL demonstrated significant electron transfer from fragment 1 to fragment 2, with a net charge transfer of 0.44–0.45e in polar solvents. In contrast, RVL B exhibited a net charge transfer of approximately 0.39e across various solvents.

**Table 4:** Apparent charge transfer (CT) and local excitation (LE) contributions percentage for the $S_0 \rightarrow S_1$ electronic transition in RVL and RVL B (as shown in Fig. 10), calculated using the Interfragment charge transfer (IFCT) method in various solvents and in the gas phase. This includes the electron transfer amount from fragment 1 ($f_{1}$) to fragment 2 ($f_{2}$) during the excitation

	RVL			RVL B		
	CT (%)	LE (%)	$f_{1}\!\rightarrow \! f_{2}$	CT (%)	LE (%)	$f_{1}\!\rightarrow \! f_{2}$
Gas	55	45	0.55e	39	61	0.39e
DCM	45	55	0.45e	39	61	0.39e
EtOH	44	56	0.44e	39	61	0.39e
ACN	44	56	0.44e	39	61	0.39e
WAT	44	56	0.44e	39	61	0.39e

**Table 5:** CAM-B3LYP functional and the 6-311++G(d,p) basis set with the solvent effect described by the PCM were used to determine the two-photon energy $\omega _{0f}$ (in eV), wavelength λ (in nm), tensor elements and TPA strength $\delta ^{TPA}$ (a.u. and GM) to RVL and RVL B in the as phase (Gas), in dichloromethane (DCM), ethanol (EtOH), acetonitrile (ACN), and water (WAT)

				Two-photon tensor elements							
Medium	State	$\omega _{0f}$	λ	S$_{xx}$	S$_{yy}$	S$_{zz}$	S$_{xy}$	S$_{xz}$	S$_{yz}$	$\delta ^{a.u.}$	$\delta ^{GM}$
RVL											
Gas	1	3.58	346.42	−36.2	6.3	−0.1	−8.6	0.0	0.0	2.43	4.61
	2	4.13	300.29	5.2	−6.4	−0.1	−3.1	0.0	0.0	0.15	0.27
	3	4.59	270.19	−0.0	−0.0	0.0	−0.0	0.5	0.1	0.00	0.00
	4	4.63	267.86	−25.3	4.6	−0.4	−2.1	−0.0	−0.0	0.00	0.00
Water	1	3.50	354.34	64.9	−19.7	0.3	13.9	−0.0	−0.0	7.22	13.69
	2	3.99	310.82	2.9	11.9	−0.2	0.2	−0.0	−0.0	0.40	0.75
	3	4.59	270.19	47.0	−12.2	0.5	2.0	0.0	0.0	6.15	11.67
	4	4.65	266.71	0.1	0.0	0.0	0.0	−1.0	−0.2	0.00	0.00
RVL B											
Gas	1	3.63	341.65	0.1	3.5	−0.3	10.8	−0.0	−0.0	0.32	0.61
	2	4.21	294.58	−22.1	6.7	0.3	−15.6	0.0	0.0	1.96	3.72
	3	4.62	268.44	12.2	−2.4	−0.3	17.9	0.0	−0.0	1.75	3.32
	4	4.65	266.71	0.0	−0.0	−0.0	0.0	−0.4	0.1	0.00	0.00
Water	1	3.54	350.34	8.1	10.8	−1.0	24.3	0.0	−0.0	1.87	3.55
	2	4.10	302.48	−22.5	13.5	0.6	−20.4	−0.0	0.0	2.56	4.86
	3	4.54	273.17	50.6	−9.7	−0.1	56.7	−0.0	−0.0	20.0	37.94
	4	5.06	245.10	204.6	−10.5	2.4	80.0	0.0	−0.0	185	351

Conversion factors: 1 a.u. = 1.896788 10$^{-50}$ cm$^{4}$ s/photon, and GM = 10$^{-50}$ cm$^{4}$ s/photon

Subsequently, the IFCT analysis was used to investigate the effect of the medium on charge transfer (CT) and the locally excited (LE) character of two hydroxy-substituted xanthone isomers present during $S_0\rightarrow S_1$ excitation. The division of the molecule into two fragments allows for a quantitative analysis of the apparent charge transfer percentage (CT%). The apparent CT% is defined as follows [45]:

1 $$\begin{aligned} CT\% = 100\% \times |\Delta p_1|, \end{aligned}$$

where $|\Delta p_1|$ denotes the variation of electron population of fragment 1, and apparent LE% is define as $100\% - CT\%$. The results in Table 4 revealed distinct electronic behaviors between the isomers. RVL exhibited a mixed LE and CT character, corresponding to an apparent CT% of 44%. In contrast, RVL B showed a predominantly LE-dominated state, with 61% LE contribution. The calculation of the electronic transition $S_0 \rightarrow S_1$ in the gas phase for RVL indicates a predominance of the CT process, with a CT contribution of 55%, indicating an alteration from the behavior observed in polar solvents. For RVL B, the behavior of the electronic transition is similar to that observed in various solvents, the charge transfer and electronic redistribution nature are insensitive to the environment.

The internal charge transfer process is closely related to the donor–acceptor group distance [46–49] and its consequence in HOMO and LUMO orbital overlap [48]. In particular, an increase in the π-conjugated chain length between these groups favors the spatial separation of the HOMO and LUMO orbitals [48, 49]. Thus, in xanthone derivatives, the predominance of the LE state arises from the close proximity of the donor and acceptor groups. Specifically, the hydroxyl at C-2 in RVL B—group with a greater contribution to the formation of the HOMO orbital **(Supplementary Material,** **Table S1)**—promotes a greater overlap between the HOMO and LUMO orbitals, as illustrated in Fig. 9, resulting in a higher contribution for the LE state.

**Table 6:** Reactivity descriptors; ΔE (energy-gap); η (hardness); ω (electrophilicity); *N* (nucleophilicity); μ (chemical potential); *I* (ionization potential); *A* (electron affinity). All values are in eV:

Components	Gas phase	Water	Gas phase	Water
$E_\text {HOMO}$ (-I)	-7.90	-7.92	-7.89	-7.91
$E_\text {LUMO}$ (-A)	-0.02	-0.67	-0.66	-0.67
ΔE	7.88	7.26	7.22	7.24
μ (chemical potential)	-3.96	-4.30	-4.28	-4.29
η (hardness)	3.94	3.63	3.61	3.62
ω (electrophilicity)	1.99	2.55	2.53	2.55
*N* (nucleophilicity)	3.09	3.07	3.10	3.08

#### Two-photon absorption analysis

Two-photon absorption (TPA) is a nonlinear process in which a molecule simultaneously absorbs two photons to reach an excited state. The efficiency of this process is quantified by the two-photon absorption cross-section ($\delta ^{\text {TPA}}$), typically expressed in Goeppert-Mayer units (GM), where $1~\text {GM} = 10^{-50}~\text {cm}^4\cdot \text {s}\cdot \text {photon}^{-1}$. The TPA transition probability for a randomly oriented molecule is given by the following [50, 51]:

2 $$\begin{aligned} \delta ^{\text {TPA}} = \frac{4\pi ^2 a_0^5 \alpha \omega ^2}{15 c_0} \left[ S_{\text {xx}}^2 + S_{\text {yy}}^2 + S_{\text {zz}}^2 + 2\left( S_{\text {xy}}^2 + S_{\text {xz}}^2 + S_{\text {yz}}^2\right) \right] , \end{aligned}$$

where $a_0$ is the Bohr radius, α is the fine structure constant, ω is the incident photon energy, $c_0$ is the speed of light, and $S_{ij}$ are the components of the two-photon transition tensor, defined as follows:

3 $$\begin{aligned} S_{ij} = \sum _{n} \left[ \frac{\langle f | \mu _i | n \rangle \langle n | \mu _j | 0 \rangle }{\omega _n - \omega } + \frac{\langle f | \mu _j | n \rangle \langle n | \mu _i | 0 \rangle }{\omega _n - \omega } \right] . \end{aligned}$$

Here, |0⟩, |n⟩, and |f⟩ represent the ground, intermediate, and final states, respectively, and $\mu _i$ is the electric dipole operator along direction *i*.

Our TPA calculations (Table 5) reveal that both compounds exhibit a solvent-dependent nonlinear response, with the TPA strength ($\delta ^{TPA}$) increasing significantly in polar media. Notably, RVL B proved to be a more efficient two-photon absorber than RVL, particularly for higher-order transitions. The S$_4$ state of RVL B in polar solvents exhibits an exceptionally high $\delta ^{TPA}$ value (up to 351 GM in water), a magnitude that is competitive when compared to established organic chromophores. For example, values varying from 36 GM and 51 GM have been reported for a series of substituted thiosemicarbazone assemblies, specially developed for nonlinear optical applications [52, 53]. In addition, this value is very close to that reported for other azo prototypes for possible photonic applications (383 GM) [51]. The pronounced performance of RVL B is intrinsically linked to the electronic character of this specific excited state, which, as suggested by the *S* tensor elements, features significant charge redistribution (supported by the IFCT analysis) that strongly favors the two-photon transition.

Our results predict that Ravenelin B, and by extension structurally related xanthones, constitute a promising class of molecules for future investigation in applications requiring two-photon absorption, such as two-photon laser scanning microscopy or photodynamic therapy.

### Global reactivity

The analysis of the global descriptors provides relevant information about the stability and reactivity of the molecules. A lower chemical potential (μ) indicates higher thermodynamic stability. On the other hand, a lower chemical hardness (η) indicates higher chemical reactivity. These parameters are easily obtained from the frontier molecular orbitals HOMO (highest occupied molecular orbital) and LUMO (lowest unoccupied molecular orbital) as follows [54]:

4 $$\begin{aligned} \mu = (E_{\text {LUMO}}+E_{\text {HOMO}})/2 \end{aligned}$$

and

5 $$\begin{aligned} \eta = (E_{\text {LUMO}} - E_{\text {HOMO}})/2 \end{aligned}$$

Table 6 shows the results obtained in vacuum and water environments. Under gaseous conditions, the chemical potential is -3.96 eV and -4.28 eV for RVL and RVL B, respectively. This means that compound RVL B is the most stable from a thermodynamic point of view. However, the solvent makes μ equivalent in both systems, leading to values of -4.30 eV and -4.29 eV.

Since the thermodynamic and, consequently, the structural stability is equivalent for both compounds, it is important to classify their chemical reactivity. RVL B is noticeably more reactive (η=3.61 eV) than RVL (η=3.94 eV) under vacuum conditions. But again, the solvent equilibrates both parameters in water, with values of 3.63 eV and 3.62 eV. In conclusion, RVL and RVL B share the same degree of stability and reactivity in a solvent environment.

Xanthones are known as potential pharmaceuticals, often used as antioxidant and antibacterial drugs. Thus, it is important to know their global electrophilicity (ω) and nucleophilicity (*N*) parameters, given as follows [54, 55]:

6 $$\begin{aligned} \omega = \mu ^2/2\eta \end{aligned}$$

and

7 $$\begin{aligned} N = E_\text {Nucleophile} - E_\text {tetracyanoethylene}. \end{aligned}$$

where *E* is the HOMO energies.

The electrophilicity (nucleophilicity) measures the propensity of a system to gain (lose) electrons, indicating what kind of target the drug will interact with in proteins and enzymes. It is possible to classify an electrophile according to the scale proposed by Domingo and collaborators, where a molecule can be classified as a marginal ($\omega \le 0.8$ eV), moderate (0.8<ω<1.5 eV), or strong ($1.5 \le \omega$) electrophile [56]. Similarly, a nucleophile can be weak (N≤1.5 eV), moderate (1.5<N<3.0 eV), or strong (3.0≤N) [57].

According to the nomenclature above, both compounds are strong electrophiles under vacuum conditions. However, RVL B gains special emphasis with ω=2.53 eV. Since the water solvent is the preferential environment for drug candidates, it is the most relevant medium; in it, both RVL and RVL B are classified as strong electrophiles with ω=2.55 eV. Concerning their nucleophilicity, both compounds are classified as strong nucleophiles with N>3.0 eV in both the gas phase and water.

At this point, two intermediate conclusions emerge. First, both compounds present an ambiphilic character, being capable of behaving as either an electrophile or a nucleophile. Thus, they can attack different targets in an enzyme. This characteristic is uncommon but quite important in pharmaceuticals. Second, although the global reactivity parameters of RVL show more sensitivity to the environment, they become equivalent to those of RVL B in solution.

**Fig. 11:**
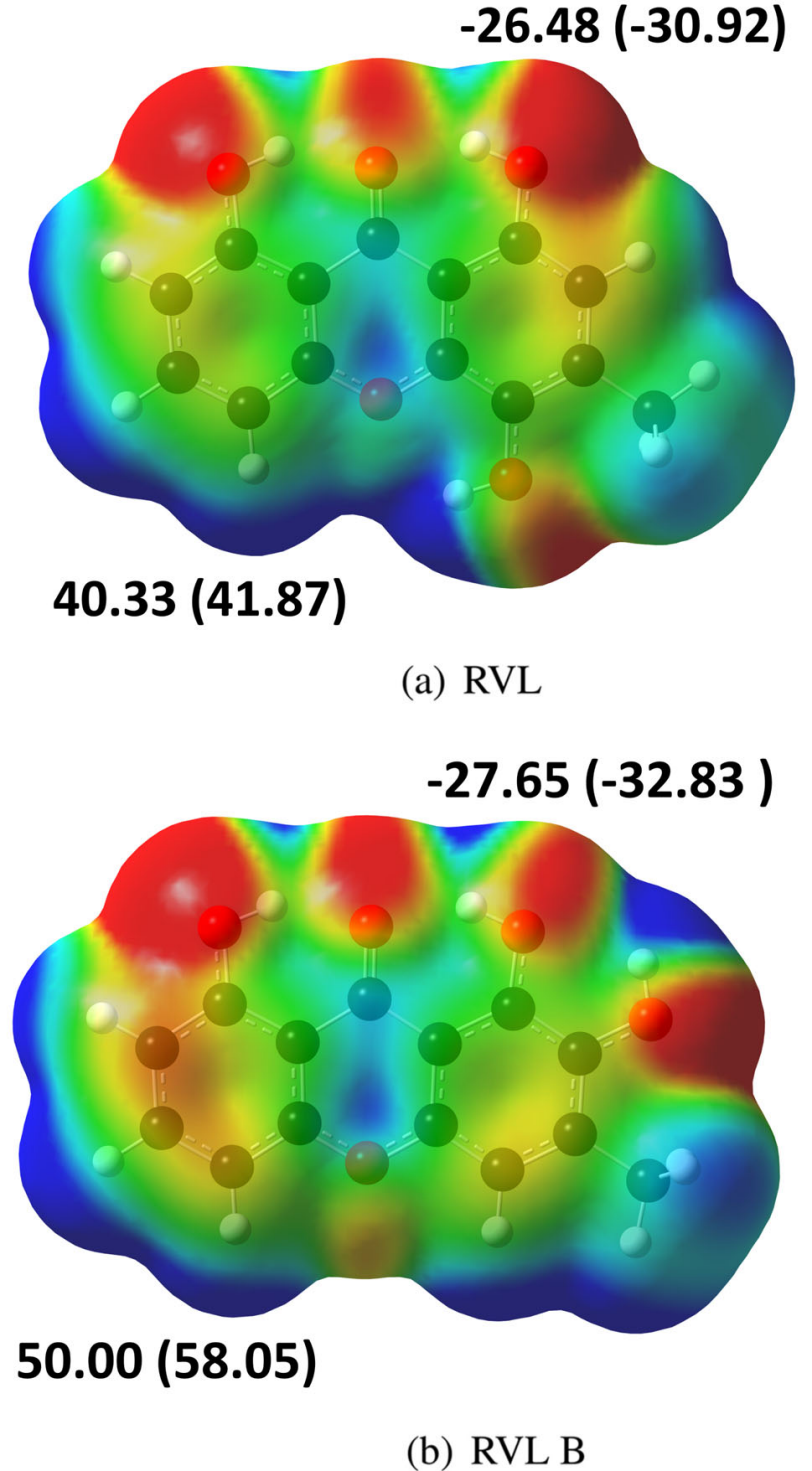
Molecular electrostatic potential (MEP) surface, at ωB97X-D/6-311++G(d,p) level calculation in gas phase

However, ω and *N* do not say anything about local reactivity, but the analysis of the molecular electrostatic potential (MEP) surface can help us to determine the preferential electrophilic and nucleophilic molecular sites [58]. Red colors indicate negative electrostatic regions, and the lowest value indicates the minimum ($V_\text {min}$), which coincides with the most nucleophilic site. On the other hand, blue colors point out positive potentials, and similarly, the highest value ($V_\text {max}$) indicates the most electrophilic site in the molecule. Analyzing Fig. 11, one observes that the $V_\text {min}$ and $V_\text {max}$ positions match for both molecules. However, RVL B presents more pronounced values in both vacuum and water solvent, possibly making it more efficient in both electrophilic and nucleophilic reactions.

## Conclusions

This study successfully demonstrates the isolation and comprehensive characterization of ravenelin B, a novel xanthone derivative from the endophytic fungus *Exserohilum rostratum*. Our integrated approach, combining experimental techniques with advanced computational methods, proved highly effective for structural elucidation of scarce natural products, overcoming the limitations imposed by minimal sample quantity and purity. The unambiguous determination of ravenelin B as 1,2,8-trihydroxy-3-methylxanthone was achieved through synergistic NMR analysis and DFT/GIAO calculations, validating this methodology as a robust solution for challenging structural characterization problems.

Beyond the structural assignment, our computational investigations revealed fundamental insights into the electronic properties of these xanthone isomers. Ravenelin B exhibits a predominant locally excited character (61% LE) in contrast to Ravenelin’s mixed charge-transfer nature, highlighting how subtle positional isomerism significantly influences electronic behavior. Particularly remarkable are the substantial two-photon absorption capabilities identified for ravenelin B, with exceptional cross-section values (up to 351 GM in water) that suggest promising applications in nonlinear optics and photodynamic therapy. The solvent-dependent photophysical behavior, including measurable Stokes shifts across different environments, enriches our understanding of the optical properties of these molecules.

The methodological framework established here provides a valuable protocol for characterizing challenging natural products when traditional approaches prove insufficient. This work not only expands the chemical diversity documented from Amazonian endophytes but also bridges natural products chemistry with materials science, revealing how computational modeling can uncover both structural and functional properties of biologically-derived molecules. Future investigations should focus on experimental validation of the predicted optical properties, biological activity assessments, and exploration of structure-activity relationships to fully realize the potential of these novel xanthone derivatives.

## Supplementary Information

Below is the link to the electronic supplementary material.

Supplementary file 1 (pdf 704 KB)

## Data Availability

No datasets were generated or analysed during the current study.
