# Supplementary material for: Discovery of ravenelin B from Exserohilum rostratum: structural elucidation of a scarce xanthone via integrated NMR/DFT-GIAO approach and comprehensive excited-state characterization
Source: J Mol Model. 2026 Jul 3;32(8):254. doi: 10.1007/s00894-026-06810-8 (PMC13331851; doi:10.1007/s00894-026-06810-8)
Supplement: Supplementary file 1 — (pdf 704 KB) [file 894_2026_6810_MOESM1_ESM.pdf]

Supplementary Material

Discovery of Ravenelin B from *Exserohilum rostratum*: Structural Elucidation of a Scarce Xanthone via Integrated NMR/DFT-GIAO Approach and Comprehensive Excited-State Characterization

Patrícia S. B. Marinho[a], Neidy S. S. dos Santos[b], Catharina B. de Araujo[c], Marcelo R. S. Siqueira[d], José E. S. Siqueira[a], Andersson Barison[e], Francinete R. Campos[f], Mayra Pinheiro[b],Rodrigo Gester[g,*], Andrey M. R. Marinho[a]

[a] Programa de Pós-Graduação em Química, Universidade Federal do Pará, Rua Augusto Corrêa, 01 – Guamá, Belém, 66075-110, Pará, Brazil.
[b] Programa de Pós-Graduação em Química, Universidade Federal do Sul e Sudeste do Pará, Marabá, 68507-590, Pará, Brazil.
[c] Instituto de Física, Universidade Federal de Alagoas (UFAL), 57072-970, Maceió, AL, Brazil.
[d] Universidade Federal do Amapá, Departamento de Ciências Exatas e Tecnológicas, Macapá, 68903-419, Amapá, Brazil.
[e] Programa de Pós-Graduação em Química, Universidade Federal do Paraná, Curitiba, 81530-900, Paraná, Brazil.
[f] Departamento de Farmácia, Universidade Federal do Paraná, Curitiba, 80060-000, Paraná, Brazil.
[g] Faculdade de Física, Universidade Federal do Sul e Sudeste do Pará, Marabá, 68507-590, Pará, Brazil.

*Corresponding author: gester@unifesspa.edu.br

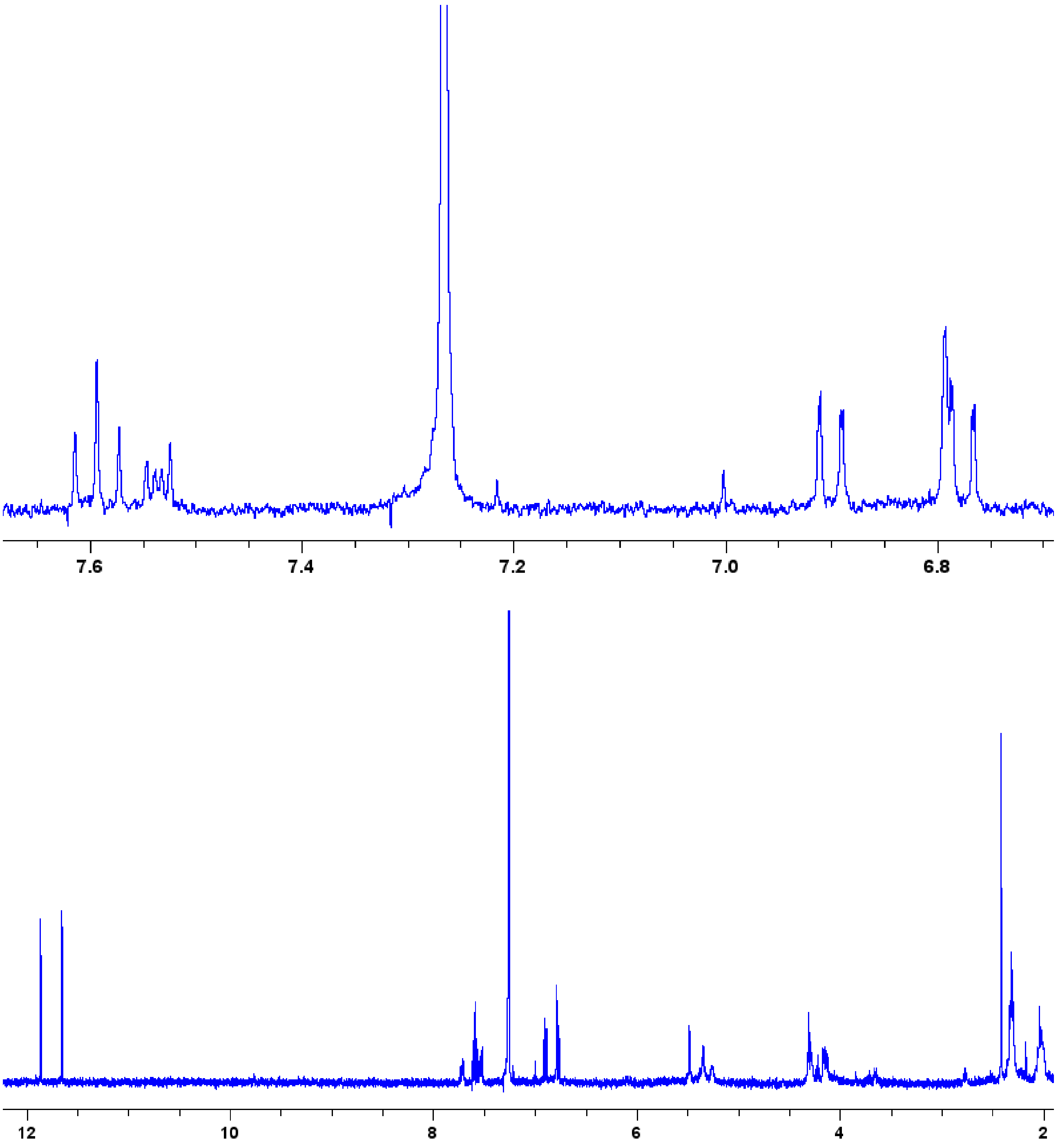

Figure S1. ^{1}H NMR spectrum of compound **RVLB** (400 MHz, $CDCl_3$).

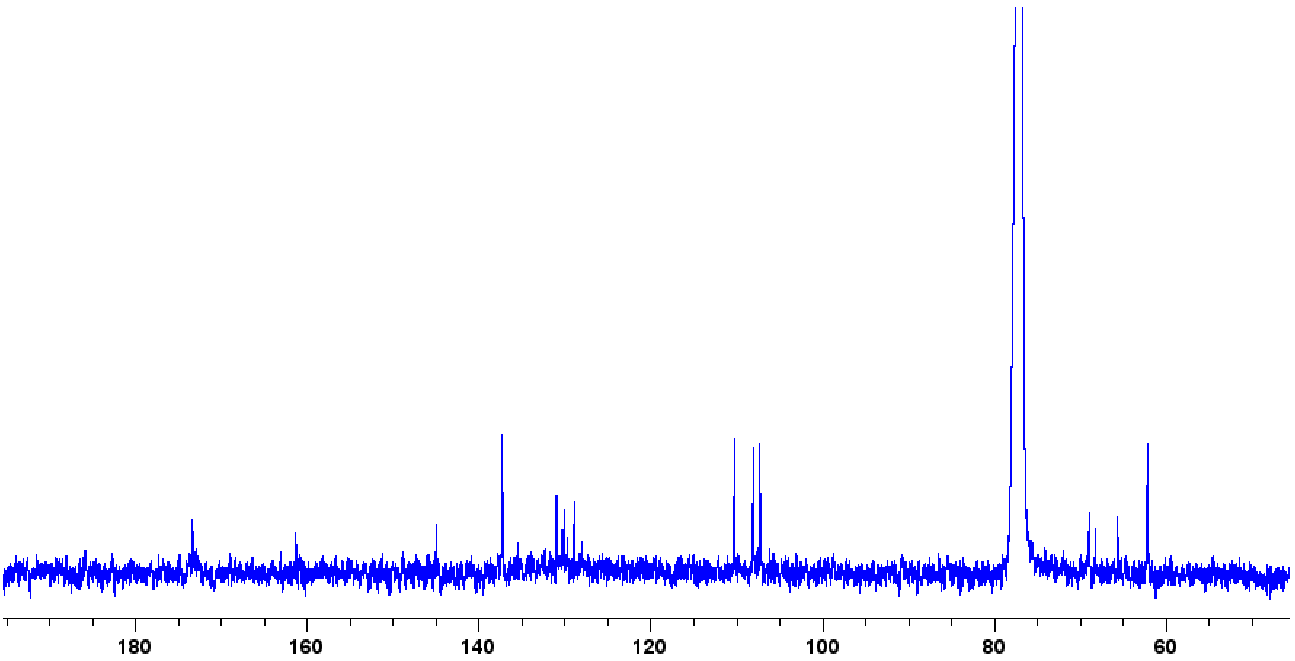

Figure S2. ^{13}C NMR spectrum of compound **RVLB** (100 MHz, $CDCl_3$).

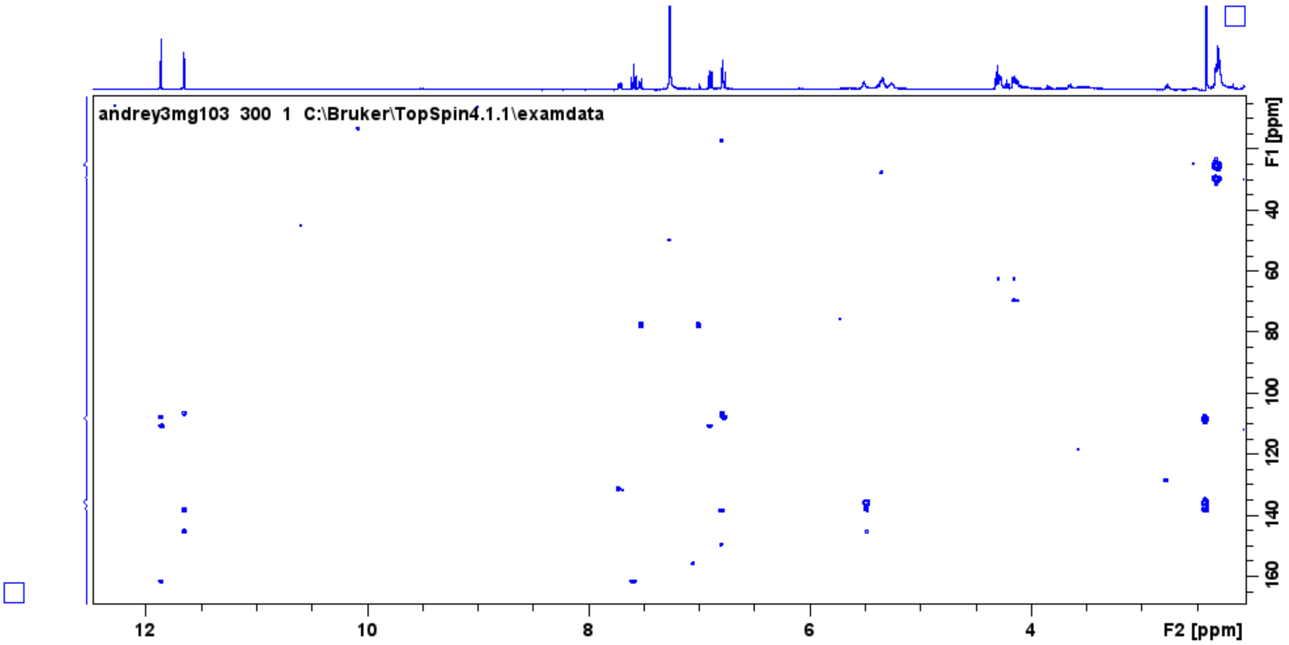

Figure S3. HMBC spectrum of compound **RVLB.**

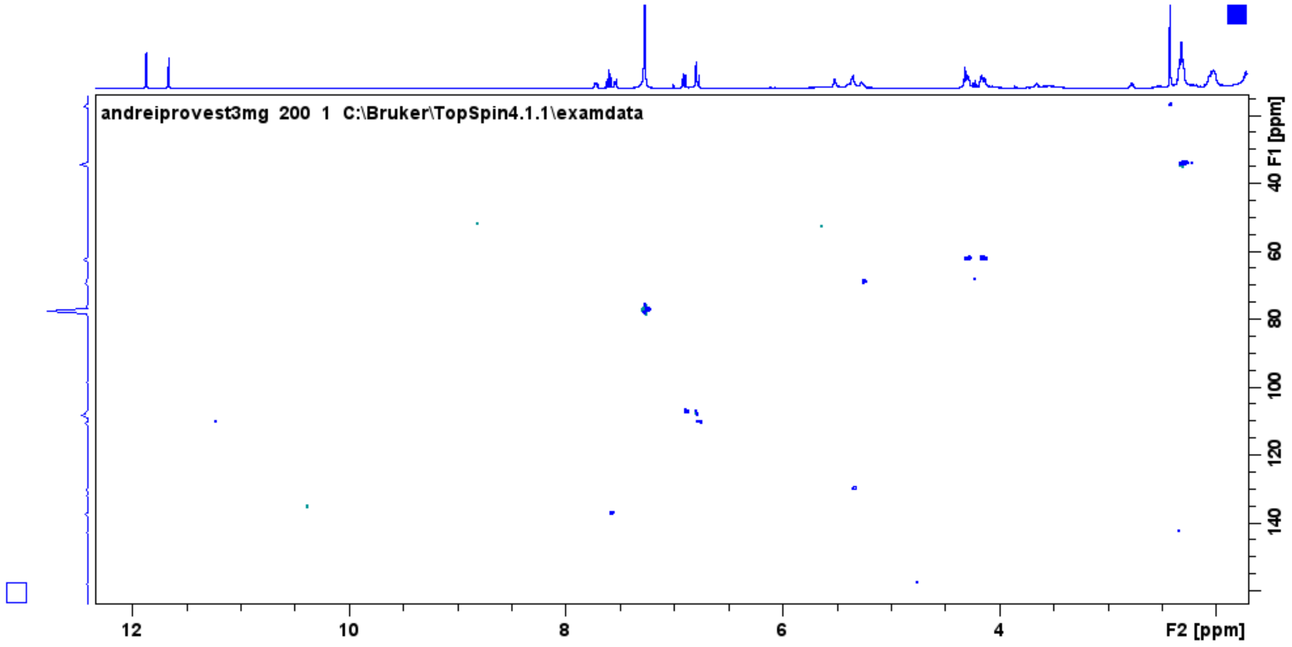

Figure S4. HSQC spectrum of compound **RVLB.**

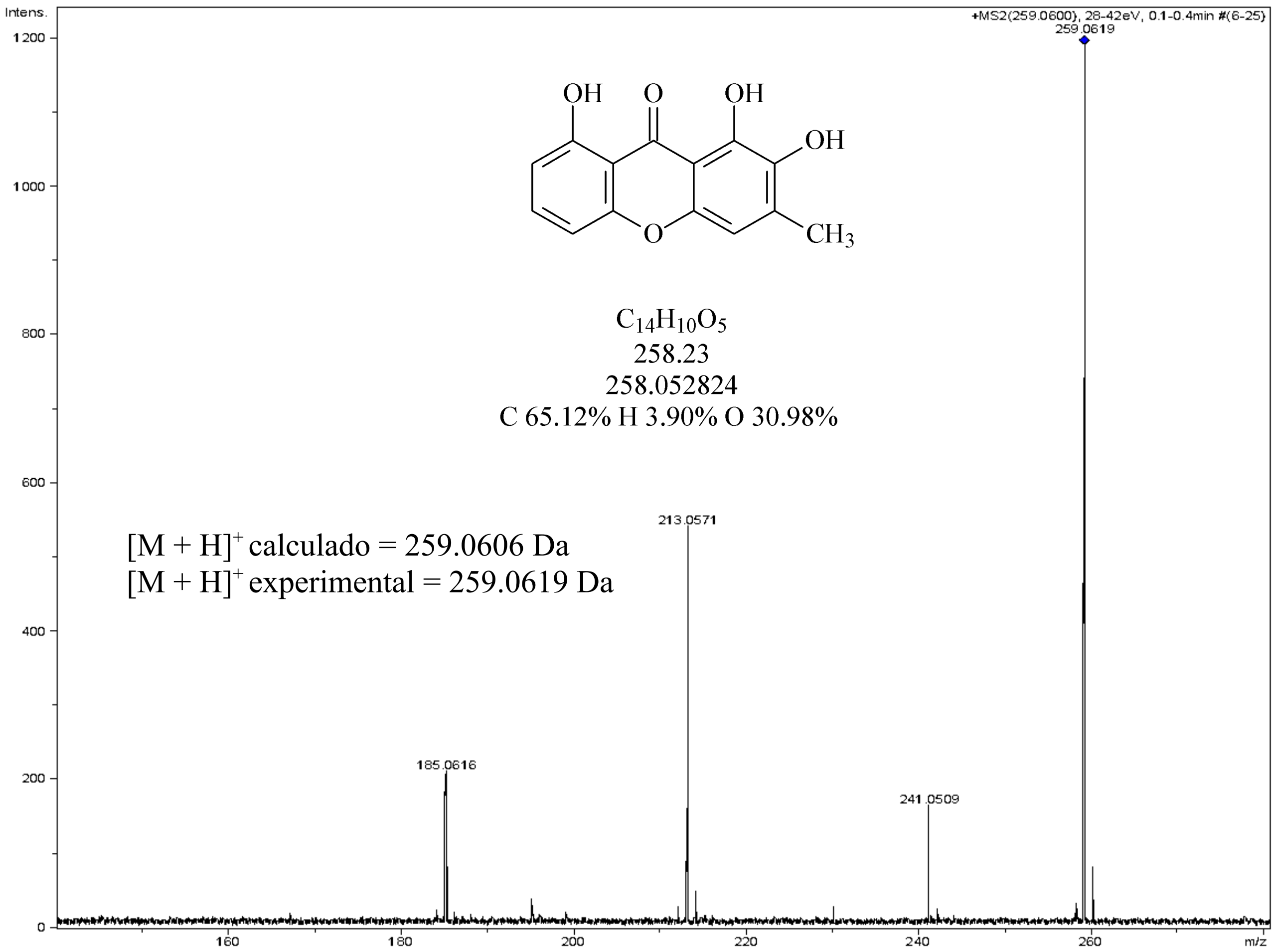

Figure S5. HR-ESIMS (full scan, positive ion mode) spectrum of compound **RVLB.**

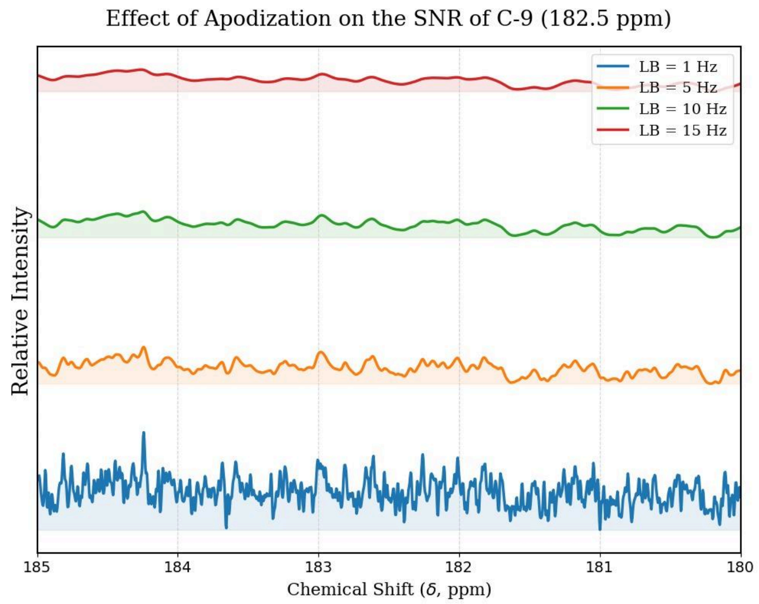

Figure S6. Apodization processing of the raw NMR data for the carbonyl region of ravenelin B (RVL B). The same spectral region is shown after applying exponential multiplication with increasing Line Broadening (LB) parameters: LB = 1.0 Hz (blue), LB = 5.0 Hz (orange), LB = 10.0 Hz (green), and LB = 15.0 Hz (red). At LB = 1.0 Hz (almost no filter), the baseline is dominated by high-frequency noise, making it impossible to distinguish a true signal from random fluctuations. As the filter strength increases (LB = 5.0 Hz), the noise is progressively suppressed, and a small plateau begins to emerge. At LB = 10.0 Hz and LB = 15.0 Hz, the noise is completely eliminated, revealing a consistent, well-defined signal centered at 182.5 ppm. This demonstrates that the baseline elevation at this position corresponds to a real physical signal (C-9 carbonyl) rather than an artifact or random noise.

Definition S1. The bond length alternation (BLA) parameters of compound 1:
$\chi_{A1} = ¼\ [r(C2-C7) + r(C6-C7) + r(C4-C5) + r(C3-C4) - 2 \cdot r(C2-C3) - 2 \cdot r(C5-C6)$
$\chi_{A2} = ¼\ [r(O2-C7) + r(O2-C8) + r(C13-C14) + r(C5-N14) - 2 \cdot r(C5-C6) - 2 \cdot r(C8-C13)$
$\chi_{A3} = ¼\ [r(C8-C9) + r(C9-C10) + r(C11-C12) + r(C12-N13) - 2 \cdot r(C8-C13) - 2 \cdot r(C10-C11)$
$\chi_{O1} = r(O1-C7) - r(O1-H5)$
$\chi_{O2} = r(O4-C7) - r(O4-H9)$
$\chi_{O3} = r(O3-C14) - r(O3-H10)$

Definition S2. The bond length alternation (BLA) parameters of compound 2:
$\chi_{A1} = ¼\ [r(C2-C7) + r(C6-C7) + r(C4-C5) + r(C3-C4) - 2 \cdot r(C2-C3) - 2 \cdot r(C5-C6)$
$\chi_{A2} = ¼\ [r(O1-C6) + r(O1-C8) + r(C13-C14) + r(C5-N14) - 2 \cdot r(C5-C6) - 2 \cdot r(C8-C13)$
$\chi_{A3} = ¼\ [r(C8-C9) + r(C9-C10) + r(C11-C12) + r(C12-N13) - 2 \cdot r(C8-C13) - 2 \cdot r(C10-C11)$
$\chi_{O1} = r(O3-C12) - r(O3-H7)$
$\chi_{O2} = r(O4-C4) - r(O4-H8)$
$\chi_{O3} = r(O5-C3) - r(O5-H9)$

Table S1. Atomic percentage contributions to the HOMO and LUMO orbitals of RVL and RVL B in gas and in acetonitrile, obtained from IFCT analysis using the Hirshfeld partition. The atomic numbering corresponds to that shown in Figure 6.

RVL			RVL B		
Atom	HOMO (%)	LUMO (%)	Atom	HOMO (%)	LUMO (%)
ACT					
C1	0.453	1.011	C1	0.310	1.067
C2	5.036	6.574	C2	3.868	7.228
C3	9.957	1.758	C3	14.408	1.822
C4	10.979	5.596	C4	9.664	5.943
C5	3.250	4.258	C5	2.260	4.239
C6	9.130	4.930	C6	8.783	4.609
C7	16.500	2.036	C7	9.167	2.435
C8	2.154	4.801	C8	2.388	4.768
C9	3.045	2.551	C9	6.129	2.588
C10	0.864	7.917	C10	1.413	7.794
C11	3.827	2.113	C11	5.801	2.060
C12	1.148	6.302	C12	2.815	6.221
C13	1.167	4.469	C13	0.662	4.341
C14	0.364	20.200	C14	0.389	19.783
O1	11.228	0.207	O1	8.907	3.433
O2	6.991	3.373	O2	0.660	13.495
O3	0.041	13.936	O3	2.301	2.413
O4	0.596	2.456	O4	6.379	2.196
O5	9.757	2.141	O5	10.135	0.073
H1	0.041	0.072	H1	0.127	0.531
H2	0.196	0.492	H2	0.127	0.531
H3	0.195	0.490	H3	0.033	0.065
H4	0.805	0.087	H4	0.524	0.191
H5	0.911	0.051	H5	0.075	1.112
H6	0.245	0.181	H6	0.510	0.132
H7	0.048	1.129	H7	0.149	0.369
H8	0.350	0.134	H8	0.432	0.353
H9	0.049	0.380	H9	0.833	0.031

H10	0.672	0.353	H10	0.752	0.179
Gas					
C1	0.422	0.998	C1	0.333	1.070
C2	4.899	6.495	C2	4.028	7.212
C3	10.252	1.766	C3	14.276	1.825
C4	11.130	5.545	C4	9.475	5.972
C5	3.824	4.166	C5	2.236	4.230
C6	9.411	4.778	C6	8.782	4.521
C7	16.825	1.905	C7	8.980	2.432
C8	1.861	4.813	C8	2.378	4.762
C9	2.460	2.572	C9	6.254	2.557
C10	0.766	8.169	C10	1.436	7.847
C11	3.181	2.267	C11	5.931	2.112
C12	0.838	6.357	C12	2.778	6.222
C13	1.144	4.550	C13	0.608	4.322
C14	0.460	19.796	C14	0.352	19.526
O1	11.353	0.173	O1	8.821	3.381
O2	6.426	3.239	O2	0.599	13.638
O3	0.085	14.126	O3	2.439	2.522
O4	0.380	2.590	O4	6.307	2.233
O5	10.821	2.247	O5	10.365	0.074
H1	0.040	0.070	H1	0.139	0.537
H2	0.178	0.491	H2	0.139	0.537
H3	0.178	0.488	H3	0.034	0.066
H4	0.830	0.088	H4	0.540	0.190
H5	0.918	0.048	H5	0.076	1.144
H6	0.198	0.186	H6	0.522	0.135
H7	0.045	1.200	H7	0.158	0.368
H8	0.294	0.146	H8	0.427	0.352
H9	0.036	0.380	H9	0.846	0.032
H10	0.745	0.350	H10	0.741	0.182

Table S2. ^{1}H and ^{13}C NMR data to compounds RVL and RVLB (400 MHz, $CDCl_3$) and theoretical chemical shifts using the calculation level B3LYP/6-311++G(d,p), with the simulations performed in solvent (Chloroform) by the IEF-PCM and GIAO approach. Chemical shifts are reported relative to tetramethylsilane (TMS). RMSD values were calculated excluding hydroxyl groups.

nuclei	RVL				RVLB			
	^{1}H		^{13}C		^{1}H		^{13}C	
	δ (Exp.)	δ (Theor.)	δ (Exp.)	δ (Theor.)	δ (Exp.)	δ (Theor.)	δ (Exp.)	δ (Theor.)
1			151.7*	160.41			144.9	150.32
2	6.64 (s)	6.81	112.2	116.03			137.8	144.54
3			136.9*	143.34			135.4	142.63
4			136.2	140.29	6.79 (q, 0.7)	6.90	108.1	111.71
4a			138.8	146.06			148.9	154.50
5	7.01 (d, 8.4 Hz)	6.97	108.2	109.80	6.90 (dd, 8.4 and 0.9)	6.97	107.2	110.46
5a			153.7	161.85			156.4	162.93
6	7.72 (t, 8.3 e 8.2 Hz)	7.74	137.5	142.91	7.59 (dd, 8.4 and 8.3)	7.79	137.2	142.81
7	6.78 (d, 8.0 Hz)	6.96	111.4	115.45	6.77 (dd, 8,3 and 0.9)	6.94	110.2	114.21
8			157.1	169.41			161.3	169.06
8a			108.4	110.94			107.6	111.17
9			187.1	190.18			182.5	190.07
9a			106.9	109.45			106.2	109.52
10	2.37 (s)	2.04; 2.55	17.1	18.68	2.42 (q, 0.7)	2.06; 2.56	16.7	18.55
0H-1	10.96 (s)	11.15			11.66 (s)	11.81		
OH-8	8.45 (sl)	12.19			11.86 (s)	11.99		
**RMSD**	**0.14**		**5.93**		**0.15**		**5.46**	

Legend: H: Hydrogen; C: Carbon; δ: Chemical shift (ppm); TMS Shielding (H): 31.8821; TMS Shielding (C): 182.4656.

Cartesian coordinates (XYZ format) of RVL in chloroform (IEF-PCM) at the B3LYP/6-311++G level (ground state).**

C	4.713930	-0.755881	0.000070
C	3.305230	-0.229290	-0.000060
C	3.043980	1.145240	-0.000220
C	1.745040	1.640910	-0.000250
C	0.653470	0.736410	-0.000140
C	0.933410	-0.642830	0.000020
C	2.234640	-1.132470	0.000070
C	-1.374220	-1.213650	0.000150
C	-2.316951	-2.232300	0.000330
C	-3.665111	-1.881040	0.000350
C	-4.079320	-0.551260	0.000190
C	-3.129300	0.467910	0.000010
C	-1.743400	0.146500	-0.000010
C	-0.722500	1.178270	-0.000190
O	2.494999	-2.476700	0.000290
O	-0.057990	-1.585890	0.000170
O	-1.023390	2.402960	-0.000170
O	-3.543560	1.747080	-0.000180
O	1.549210	2.977890	-0.000430
H	5.435320	0.061269	-0.001630
H	4.895750	-1.381601	0.878590
H	4.894860	-1.384631	-0.876430
H	3.866580	1.850090	-0.000350
H	1.659399	-2.961530	0.000150
H	-1.995191	-3.265170	0.000450
H	-4.412521	-2.665899	0.000480
H	-5.129600	-0.289279	0.000200
H	-2.740770	2.320480	-0.000350
H	0.578650	3.134430	-0.000490

Cartesian coordinates (XYZ format) of RVL in chloroform (IEF-PCM) at the ωB97X-D/6-311++G level (ground state).**

C	4.693520	-0.766105	-0.000011
C	3.291101	-0.232114	-0.000101
C	3.037222	1.138977	-0.000281
C	1.742852	1.637298	-0.000251
C	0.654451	0.738129	-0.000061
C	0.927180	-0.636782	0.000119
C	2.222930	-1.129813	0.000129
C	-1.365650	-1.204780	0.000209
C	-2.302831	-2.224029	0.000309
C	-3.647681	-1.877198	0.000229
C	-4.065270	-0.552848	0.000069
C	-3.120309	0.466362	-0.000021
C	-1.738879	0.148640	0.000039
C	-0.720738	1.182990	-0.000071
O	2.476859	-2.466763	0.000369
O	-0.058060	-1.573421	0.000269
O	-1.017967	2.397090	-0.000341
O	-3.539528	1.735512	-0.000141
O	1.555513	2.967598	-0.000411
H	5.420051	0.046425	-0.002031
H	4.867410	-1.391505	0.879539
H	4.866270	-1.395015	-0.877251
H	3.862302	1.841296	-0.000481
H	1.643439	-2.947132	0.000239
H	-1.977182	-3.255669	0.000429
H	-4.392891	-2.664297	0.000299
H	-5.116289	-0.293817	0.000009
H	-2.748157	2.313401	-0.000141
H	0.593113	3.133829	-0.000361

Cartesian coordinates (XYZ format) of RVL in the gas phase at the CAM-B3LYP/6-311++G level (ground state).**

C	4.690111	-0.761497	0.000050
C	3.290131	-0.225368	-0.000070
C	3.033290	1.141652	-0.000220
C	1.737870	1.636851	-0.000240
C	0.654200	0.734500	-0.000120
C	0.931171	-0.638500	0.000050
C	2.224551	-1.124919	0.000080
C	-1.368569	-1.208761	0.000160
C	-2.307838	-2.222141	0.000330
C	-3.650978	-1.870722	0.000330
C	-4.063249	-0.548402	0.000160
C	-3.115570	0.467748	-0.000010
C	-1.737439	0.143989	0.000000
C	-0.718990	1.176660	-0.000170
O	2.484082	-2.463969	0.000300
O	-0.057208	-1.579920	0.000190
O	-1.017891	2.390129	-0.000200
O	-3.528630	1.735768	-0.000190
O	1.546149	2.963421	-0.000420
H	5.418471	0.048333	-0.001680
H	4.862391	-1.389587	0.876860
H	4.861441	-1.392617	-0.874740
H	3.852010	1.849772	-0.000370
H	1.649522	-2.945269	0.000180
H	-1.985188	-3.254021	0.000460
H	-4.398288	-2.655203	0.000450
H	-5.110959	-0.279853	0.000160
H	-2.734831	2.316318	-0.000330
H	0.581279	3.132160	-0.000460

Cartesian coordinates (XYZ format) of RVL in the gas phase at the CAM-B3LYP/6-311++G level (emission state).**

C	4.749794	-0.730877	0.000025
C	3.348470	-0.200275	-0.000049
C	3.049062	1.144310	-0.000265
C	1.705835	1.614654	-0.000285
C	0.626944	0.705588	-0.000108
C	0.913880	-0.627077	0.000112
C	2.261705	-1.100117	0.000152
C	-1.382851	-1.213325	0.000254
C	-2.301910	-2.232937	0.000452
C	-3.656081	-1.902985	0.000402
C	-4.060287	-0.569966	0.000153
C	-3.127347	0.451539	-0.000048
C	-1.737022	0.154717	0.000001
C	-0.747763	1.164329	-0.000194
O	2.490270	-2.408069	0.000377
O	-0.025705	-1.582497	0.000296
O	-0.981462	2.446080	-0.000480
O	-3.536647	1.732281	-0.000294
O	1.447083	2.893116	-0.000452
H	5.468070	0.087875	-0.000559
H	4.935887	-1.353409	0.878298
H	4.935600	-1.354432	-0.877577
H	3.836860	1.887253	-0.000417
H	1.643897	-2.881504	0.000483
H	-1.966928	-3.261701	0.000639
H	-4.398508	-2.689874	0.000555
H	-5.109597	-0.304031	0.000109
H	-2.742019	2.311234	-0.000418
H	0.393858	2.965827	-0.000305

Cartesian coordinates (XYZ format) of RVL in water (IEF-PCM) at the CAM-B3LYP/6-311++G level (ground state).**

C	4.691809	-0.758431	0.000079
C	3.289940	-0.227440	-0.000061
C	3.032540	1.141620	-0.000221
C	1.738420	1.634580	-0.000251
C	0.653710	0.733950	-0.000141
C	0.929700	-0.638720	0.000019
C	2.224629	-1.125870	0.000069
C	-1.367481	-1.208030	0.000149
C	-2.305951	-2.222910	0.000329
C	-3.648681	-1.873100	0.000349
C	-4.062310	-0.549110	0.000199
C	-3.115800	0.465460	0.000009
C	-1.737150	0.144760	-0.000011
C	-0.719300	1.175560	-0.000201
O	2.481059	-2.465380	0.000289
O	-0.056941	-1.577480	0.000169
O	-1.016900	2.391470	-0.000161
O	-3.527660	1.738930	-0.000181
O	1.541530	2.965350	-0.000431
H	5.416470	0.054419	-0.001601
H	4.867769	-1.384261	0.877819
H	4.866889	-1.387241	-0.875681
H	3.854240	1.846500	-0.000351
H	1.647049	-2.950110	0.000149
H	-1.982621	-3.254620	0.000449
H	-4.395461	-2.657790	0.000489
H	-5.111570	-0.285910	0.000209
H	-2.730200	2.315390	-0.000351
H	0.574290	3.126600	-0.000491

Cartesian coordinates (XYZ format) of RVL in water (IEF-PCM) at the CAM-B3LYP/6-311++G level (emission state).**

C	4.743416	-0.736428	0.000138
C	3.346523	-0.201849	-0.000011
C	3.051312	1.141545	-0.000248
C	1.714927	1.611487	-0.000357
C	0.617987	0.709764	-0.000179
C	0.911923	-0.634971	0.000034
C	2.247589	-1.106507	0.000119
C	-1.381619	-1.215014	0.000176
C	-2.310039	-2.233569	0.000360
C	-3.660148	-1.896581	0.000379
C	-4.061452	-0.563511	0.000224
C	-3.117090	0.451127	0.000043
C	-1.738101	0.147460	0.000007
C	-0.729781	1.163409	-0.000177
O	2.494340	-2.405846	0.000341
O	-0.043587	-1.584503	0.000193
O	-1.000033	2.448117	-0.000220
O	-3.516762	1.744299	-0.000102
O	1.457463	2.899385	-0.000675
H	5.462571	0.081036	-0.000359
H	4.926878	-1.359330	0.879185
H	4.926713	-1.360277	-0.878265
H	3.844350	1.878862	-0.000393
H	1.665862	-2.911596	0.000410
H	-1.977912	-3.263147	0.000489
H	-4.406377	-2.680370	0.000522
H	-5.111517	-0.298923	0.000244
H	-2.699779	2.302163	-0.000221
H	0.425154	2.981791	-0.000953

Cartesian coordinates (XYZ format) of RVL in the gas phase at the ωB97X-D/6-311++G level (ground state).**

C	4.691698	-0.768999	-0.000013
C	3.291129	-0.230123	-0.000116
C	3.037858	1.139038	-0.000245
C	1.742419	1.639526	-0.000197
C	0.654994	0.738588	-0.000053
C	0.928615	-0.636547	0.000075
C	2.222891	-1.128770	0.000077
C	-1.366637	-1.205561	0.000159
C	-2.304755	-2.223231	0.000258
C	-3.649826	-1.874707	0.000217
C	-4.065957	-0.551939	0.000081
C	-3.119837	0.468644	-0.000017
C	-1.738956	0.147744	0.000025
C	-0.720224	1.183819	-0.000067
O	2.479082	-2.465723	0.000290
O	-0.058208	-1.575901	0.000206
O	-1.019010	2.395697	-0.000157
O	-3.539658	1.732773	-0.000168
O	1.559481	2.965669	-0.000331
H	5.421713	0.040732	-0.002643
H	4.862267	-1.396036	0.878974
H	4.860757	-1.400585	-0.875992
H	3.860109	1.844386	-0.000426
H	1.644415	-2.941469	0.000093
H	-1.979870	-3.255101	0.000365
H	-4.395734	-2.661474	0.000291
H	-5.115415	-0.287587	0.000044
H	-2.751331	2.314035	-0.000237
H	0.599118	3.138073	-0.000309

Cartesian coordinates (XYZ format) of RVL in the gas phase at the ωB97X-D/6-311++G level (emission state).**

C	4.750366	-0.745673	0.000069
C	3.350303	-0.209647	-0.000056
C	3.054495	1.138011	-0.000258
C	1.713800	1.614216	-0.000295
C	0.624150	0.713915	-0.000115
C	0.910331	-0.623995	0.000074
C	2.254865	-1.104077	0.000121
C	-1.381900	-1.207573	0.000207
C	-2.300721	-2.233515	0.000381
C	-3.654772	-1.904689	0.000361
C	-4.062650	-0.572992	0.000169
C	-3.128214	0.453235	-0.000006
C	-1.739565	0.156646	0.000011
C	-0.744886	1.177648	-0.000162
O	2.478727	-2.411494	0.000337
O	-0.030892	-1.577954	0.000235
O	-0.989568	2.452789	-0.000340
O	-3.544486	1.728687	-0.000189
O	1.472611	2.898817	-0.000511
H	5.471428	0.072039	-0.000867
H	4.933902	-1.368195	0.880139
H	4.933448	-1.369844	-0.878915
H	3.846800	1.877543	-0.000414
H	1.631660	-2.877488	0.000421
H	-1.963439	-3.262198	0.000529
H	-4.396644	-2.693539	0.000497
H	-5.113445	-0.310182	0.000152
H	-2.751895	2.306369	-0.000296
H	0.443433	2.989656	-0.000521

Cartesian coordinates (XYZ format) of RVL in water (IEF-PCM) at the ωB97X-D/6-311++G level (ground state).**

C	4.694002	-0.765040	-0.000023
C	3.290784	-0.232985	-0.000111
C	3.036881	1.139160	-0.000257
C	1.743121	1.636660	-0.000213
C	0.654134	0.738062	-0.000060
C	0.926261	-0.636727	0.000089
C	2.222897	-1.130235	0.000097
C	-1.364916	-1.204639	0.000182
C	-2.301793	-2.224681	0.000285
C	-3.646343	-1.878417	0.000231
C	-4.064573	-0.553179	0.000078
C	-3.120175	0.465434	-0.000024
C	-1.738611	0.148740	0.000031
C	-0.721132	1.182616	-0.000069
O	2.475393	-2.467196	0.000340
O	-0.057872	-1.572184	0.000234
O	-1.018823	2.397454	-0.000213
O	-3.538539	1.736894	-0.000198
O	1.553896	2.968521	-0.000351
H	5.419330	0.048453	-0.002354
H	4.869472	-1.389266	0.880120
H	4.868153	-1.393316	-0.877509
H	3.863384	1.839931	-0.000446
H	1.642430	-2.949388	0.000143
H	-1.975535	-3.256119	0.000401
H	-4.391460	-2.665464	0.000305
H	-5.116247	-0.296456	0.000029
H	-2.745873	2.313268	-0.000270
H	0.590659	3.131819	-0.000324

Cartesian coordinates (XYZ format) of RVL in water (IEF-PCM) at the ωB97X-D/6-311++G level (emission state).**

C	4.744234	-0.749453	0.000029
C	3.348308	-0.209786	-0.000094
C	3.056333	1.136751	-0.000279
C	1.722450	1.613004	-0.000270
C	0.616557	0.717635	-0.000083
C	0.908894	-0.632022	0.000112
C	2.242763	-1.110499	0.000124
C	-1.380533	-1.210077	0.000247
C	-2.307889	-2.234444	0.000417
C	-3.658736	-1.899380	0.000360
C	-4.064590	-0.566847	0.000135
C	-3.120317	0.452657	-0.000035
C	-1.740860	0.150516	0.000023
C	-0.728586	1.175115	-0.000153
O	2.485910	-2.409103	0.000325
O	-0.047108	-1.580061	0.000284
O	-1.006718	2.452842	-0.000441
O	-3.527534	1.740247	-0.000254
O	1.481203	2.905247	-0.000420
H	5.466786	0.066440	-0.001056
H	4.925242	-1.372283	0.880858
H	4.924690	-1.374196	-0.879541
H	3.852663	1.872060	-0.000437
H	1.657819	-2.909155	0.000453
H	-1.973503	-3.263945	0.000583
H	-4.403579	-2.685762	0.000488
H	-5.116234	-0.305890	0.000087
H	-2.714772	2.298447	-0.000343
H	0.466685	3.001883	-0.000239

Cartesian coordinates (XYZ format) of RVL in the gas phase at the M06-HF/6-311++G level (ground state).**

C	4.692291	-0.776737	0.000070
C	3.287010	-0.221158	-0.000090
C	3.036959	1.147651	-0.000250
C	1.739339	1.640890	-0.000260
C	0.662010	0.734920	-0.000120
C	0.929381	-0.638180	0.000050
C	2.224811	-1.122779	0.000080
C	-1.366339	-1.210002	0.000170
C	-2.306178	-2.225193	0.000320
C	-3.650418	-1.871494	0.000320
C	-4.065629	-0.547744	0.000160
C	-3.116340	0.467897	0.000010
C	-1.741910	0.139138	0.000010
C	-0.720761	1.181669	-0.000150
O	2.482232	-2.458919	0.000320
O	-0.057049	-1.574801	0.000190
O	-1.017832	2.379398	-0.000250
O	-3.524051	1.735707	-0.000150
O	1.543768	2.965660	-0.000430
H	5.416820	0.033343	-0.001750
H	4.837481	-1.400297	0.881630
H	4.836521	-1.403497	-0.879350
H	3.852109	1.856832	-0.000400
H	1.658352	-2.953630	0.000110
H	-1.980547	-3.253762	0.000450
H	-4.395688	-2.654444	0.000440
H	-5.110210	-0.276005	0.000160
H	-2.746122	2.327317	-0.000250
H	0.589378	3.154510	-0.000440

Cartesian coordinates (XYZ format) of RVL in the gas phase at the M06-HF/6-311++G level (emission state).**

C	4.753382	-0.726365	-0.000051
C	3.347055	-0.180521	0.000018
C	3.049211	1.169661	0.000023
C	1.704476	1.635273	-0.000008
C	0.607874	0.695839	0.000157
C	0.914536	-0.646156	-0.000003
C	2.256594	-1.094401	0.000043
C	-1.374999	-1.227119	0.000003
C	-2.310255	-2.239825	-0.000019
C	-3.658594	-1.887406	-0.000030
C	-4.059706	-0.556710	-0.000022
C	-3.110262	0.457887	-0.000000
C	-1.737543	0.133027	0.000030
C	-0.719234	1.151836	0.000040
O	2.512344	-2.397606	0.000000
O	-0.040214	-1.597572	-0.000005
O	-0.973984	2.431908	-0.000042
O	-3.503641	1.737308	0.000012
O	1.395250	2.881959	-0.000061
H	5.464826	0.095779	0.000673
H	4.917142	-1.346296	0.881613
H	4.917525	-1.345060	-0.882523
H	3.831881	1.915660	-0.000012
H	1.693464	-2.908120	0.000030
H	-1.985988	-3.269235	-0.000034
H	-4.406764	-2.666524	-0.000050
H	-5.103563	-0.280196	-0.000035
H	-2.715905	2.321332	-0.000003
H	0.294132	2.924560	0.000013

Cartesian coordinates (XYZ format) of RVL in water (IEF-PCM) at the M06-HF/6-311++G level (ground state).**

C	4.695219	-0.772671	0.000101
C	3.287139	-0.223351	-0.000089
C	3.036429	1.147989	-0.000259
C	1.739969	1.638819	-0.000279
C	0.661519	0.733919	-0.000139
C	0.927859	-0.638721	0.000031
C	2.225289	-1.124101	0.000071
C	-1.365661	-1.209591	0.000171
C	-2.304851	-2.226431	0.000321
C	-3.648671	-1.873841	0.000331
C	-4.065311	-0.547961	0.000181
C	-3.117091	0.465879	0.000021
C	-1.741881	0.139649	0.000011
C	-0.721291	1.180319	-0.000159
O	2.479949	-2.460431	0.000321
O	-0.056901	-1.572471	0.000181
O	-1.016530	2.380259	-0.000259
O	-3.522721	1.739679	-0.000119
O	1.538250	2.967519	-0.000449
H	5.414909	0.041408	-0.001679
H	4.844739	-1.393911	0.882631
H	4.843849	-1.397071	-0.880319
H	3.855219	1.853409	-0.000399
H	1.657269	-2.960051	0.000081
H	-1.979181	-3.255261	0.000441
H	-4.393701	-2.656890	0.000451
H	-5.111821	-0.282560	0.000191
H	-2.740490	2.326879	-0.000229
H	0.580820	3.148169	-0.000449

Cartesian coordinates (XYZ format) of RVL in water (IEF-PCM) at the M06-HF/6-311++G level (emission state).**

C	4.749779	-0.721109	0.000077
C	3.344657	-0.175862	-0.000079
C	3.052157	1.172227	-0.000275
C	1.709945	1.634329	-0.000309
C	0.602878	0.691805	-0.000107
C	0.912565	-0.660360	0.000080
C	2.246719	-1.101284	0.000120
C	-1.376066	-1.232606	0.000220
C	-2.320365	-2.241423	0.000401
C	-3.665104	-1.881374	0.000381
C	-4.062900	-0.546130	0.000183
C	-3.105743	0.458874	-0.000003
C	-1.737487	0.127976	0.000017
C	-0.708393	1.143845	-0.000168
O	2.524737	-2.394315	0.000336
O	-0.052818	-1.604240	0.000244
O	-0.981292	2.432244	-0.000373
O	-3.482216	1.753555	-0.000206
O	1.390649	2.882232	-0.000511
H	5.458896	0.102548	-0.000709
H	4.913866	-1.338985	0.883483
H	4.913535	-1.340407	-0.882382
H	3.839477	1.913773	-0.000425
H	1.726027	-2.938833	0.000451
H	-2.001804	-3.272985	0.000555
H	-4.417627	-2.656501	0.000524
H	-5.108222	-0.273259	0.000166
H	-2.671237	2.314200	-0.000334
H	0.298753	2.921204	-0.000467

Cartesian coordinates (XYZ format) of RVL B in chloroform (IEF-PCM) at the B3LYP/6-311++G level (ground state).**

C	-4.409658	-1.732981	0.000150
C	-3.080498	-1.029941	0.000040
C	-3.043099	0.372519	0.000120
C	-1.827119	1.051410	0.000010
C	-0.609699	0.334080	-0.000150
C	-0.667058	-1.071650	-0.000220
C	-1.879718	-1.745280	-0.000150
C	1.700842	-1.237220	-0.000220
C	2.804652	-2.082879	-0.000130
C	4.073912	-1.512429	0.000070
C	4.262671	-0.130639	0.000160
C	3.157531	0.714921	0.000030
C	1.843671	0.166720	-0.000140
C	0.667491	1.009480	-0.000220
O	0.473082	-1.827410	-0.000280
O	0.747251	2.270000	0.000610
O	3.350431	2.046601	-0.000060
O	-1.872199	2.404330	-0.000070
O	-4.219139	1.069279	0.000270
H	-4.998648	-1.451021	0.877640
H	-4.998908	-1.450801	-0.877090
H	-4.279798	-2.815351	0.000000
H	2.658782	-3.154809	-0.000170
H	4.941862	-2.161639	0.000150
H	5.255171	0.301411	0.000270
H	2.463131	2.476291	-0.000400
H	-0.941349	2.729770	-0.000420
H	-4.014729	2.014929	0.000300
H	-1.884448	-2.827840	-0.000190

Cartesian coordinates (XYZ format) of RVL B in chloroform (IEF-PCM) at the ωB97X-D/6-311++G level (ground state).**

C	-4.393140	-1.728170	0.000040
C	-3.066540	-1.027270	0.000030
C	-3.034820	0.369880	0.000040
C	-1.823790	1.049420	0.000040
C	-0.609690	0.335600	0.000030
C	-0.661310	-1.064130	0.000020
C	-1.869400	-1.739150	0.000020
C	1.691690	-1.229521	-0.000020
C	2.790570	-2.076311	-0.000050
C	4.057090	-1.510421	-0.000090
C	4.250010	-0.133471	-0.000080
C	3.149570	0.712619	-0.000050
C	1.839720	0.168059	-0.000010
C	0.666110	1.014079	0.000020
O	0.471790	-1.815511	-0.000000
O	0.745741	2.263159	-0.000030
O	3.348031	2.035169	-0.000030
O	-1.876669	2.393940	0.000060
O	-4.206360	1.058720	0.000050
H	-4.978490	-1.443520	0.878270
H	-4.978570	-1.443400	-0.878110
H	-4.263780	-2.810560	-0.000040
H	2.641060	-3.147721	-0.000060
H	4.923320	-2.162141	-0.000120
H	5.243450	0.296349	-0.000110
H	2.471211	2.471569	0.000020
H	-0.956749	2.729290	0.000080
H	-4.006719	2.001010	0.000050
H	-1.871430	-2.821970	0.000020

Cartesian coordinates (XYZ format) of RVL B in the gas phase at the CAM-B3LYP/6-311++G level (ground state).**

C	-4.395601	-1.717388	0.000110
C	-3.065780	-1.026108	0.000040
C	-3.029780	0.368282	0.000090
C	-1.819489	1.043591	0.000010
C	-0.609180	0.328301	-0.000080
C	-0.663200	-1.069699	-0.000130
C	-1.871361	-1.740319	-0.000080
C	1.694819	-1.231780	-0.000140
C	2.794929	-2.072251	-0.000110
C	4.058199	-1.501292	0.000000
C	4.246250	-0.126602	0.000060
C	3.144020	0.715439	0.000010
C	1.838340	0.164960	-0.000090
C	0.664481	1.006080	-0.000110
O	0.473289	-1.820540	-0.000170
O	0.740071	2.256020	0.000370
O	3.335601	2.036669	-0.000030
O	-1.868479	2.389101	-0.000040
O	-4.198149	1.064212	0.000180
O	-4.980191	-1.426817	0.875750
H	-4.980381	-1.426647	-0.875330
H	-4.275231	-2.800118	0.000000
H	2.646719	-3.143061	-0.000140
H	4.926349	-2.149682	0.000040
H	5.235160	0.311378	0.000130
H	2.456351	2.474909	-0.000210
H	-0.943839	2.724251	-0.000270
H	-3.989279	2.007042	0.000190
H	-1.874211	-2.822239	-0.000120

Cartesian coordinates (XYZ format) of RVL B in the gas phase at the CAM-B3LYP/6-311++G level (emission state).**

C	-4.461352	-1.710385	-0.000172
C	-3.125229	-1.032589	-0.000081
C	-3.051869	0.375729	-0.000227
C	-1.805645	1.108693	-0.000191
C	-0.605246	0.358223	0.000383
C	-0.689115	-1.011359	0.000408
C	-1.936315	-1.713291	0.000246
C	1.670283	-1.228587	0.000175
C	2.726010	-2.108462	-0.000189
C	4.019588	-1.597829	-0.000527
C	4.235018	-0.222829	-0.000540
C	3.174539	0.661832	-0.000253
C	1.834122	0.179377	0.000175
C	0.689738	0.984076	0.000580
O	0.399745	-1.786630	0.000712
O	0.784384	2.333941	0.001476
O	3.445672	1.985530	-0.000545
O	-1.861526	2.371475	-0.000571
O	-4.144350	1.119051	-0.000624
H	-5.043749	-1.421985	0.877611
H	-5.044335	-1.420645	-0.877111
H	-4.346398	-2.793676	-0.001009
H	2.529211	-3.171953	-0.000153
H	4.864326	-2.273726	-0.000795
H	5.236803	0.187005	-0.000832
H	2.620621	2.494321	-0.000048
H	-0.130049	2.706010	0.000547
H	-3.821949	2.046698	-0.000817
H	-1.903032	-2.794576	0.000310

Cartesian coordinates (XYZ format) of RVL B in water (IEF-PCM) at the CAM-B3LYP/6-311++G level (ground state).**

C	-4.393170	-1.722461	0.000160
C	-3.065860	-1.026300	0.000040
C	-3.029821	0.369020	0.000130
C	-1.820301	1.044700	0.000010
C	-0.609051	0.329921	-0.000160
C	-0.663370	-1.068039	-0.000230
C	-1.871170	-1.739730	-0.000150
C	1.694670	-1.231419	-0.000220
C	2.794730	-2.072968	-0.000130
C	4.058100	-1.503498	0.000080
C	4.246069	-0.127208	0.000170
C	3.144279	0.712952	0.000030
C	1.838129	0.164962	-0.000150
C	0.664559	1.005961	-0.000240
O	0.472780	-1.819009	-0.000290
O	0.742338	2.257181	0.000610
O	3.333369	2.039032	-0.000050
O	-1.866662	2.391490	-0.000070
O	-4.200531	1.064769	0.000280
H	-4.978550	-1.436671	0.876920
H	-4.978820	-1.436451	-0.876340
H	-4.267530	-2.804241	0.000010
H	2.648231	-3.144148	-0.000180
H	4.926000	-2.151817	0.000170
H	5.236919	0.306843	0.000290
H	2.450298	2.471832	-0.000390
H	-0.941022	2.723920	-0.000420
H	-3.997721	2.009379	0.000310
H	-1.874920	-2.821710	-0.000200

Cartesian coordinates (XYZ format) of RVL B in water (IEF-PCM) at the CAM-B3LYP/6-311++G level (emission state).**

C	-4.454129	-1.698597	0.000038
C	-3.116690	-1.026006	0.000029
C	-3.033061	0.382903	0.000048
C	-1.774080	1.054383	0.000039
C	-0.576415	0.329434	0.000011
C	-0.673549	-1.056997	-0.000005
C	-1.920634	-1.721866	0.000004
C	1.691279	-1.233453	-0.000037
C	2.771191	-2.090381	-0.000057
C	4.055171	-1.544677	-0.000065
C	4.239459	-0.168975	-0.000056
C	3.139770	0.684282	-0.000039
C	1.833044	0.165451	-0.000027
C	0.669160	0.999522	0.000001
O	0.434000	-1.818249	-0.000028
O	0.716280	2.314705	0.000027
O	3.331641	2.023009	-0.000043
O	-1.752631	2.370210	0.000059
O	-4.143375	1.104123	0.000074
H	-5.038549	-1.415580	0.879105
H	-5.038568	-1.415566	-0.879010
H	-4.336622	-2.781269	0.000028
H	2.606513	-3.159611	-0.000064
H	4.914851	-2.201815	-0.000080
H	5.233018	0.261577	-0.000066
H	2.439975	2.448097	-0.000050
H	-0.728914	2.617597	0.000047
H	-3.925549	2.050680	0.000083
H	-1.916575	-2.804629	-0.000010

Cartesian coordinates (XYZ format) of RVL B in the gas phase at the ωB97X-D/6-311++G level (ground state).**

C	4.386280	-1.750814	0.000062
C	3.072778	-1.018831	-0.000114
C	3.040088	0.376008	-0.000145
C	1.821455	1.050211	-0.000149
C	0.610711	0.336344	-0.000048
C	0.664987	-1.064588	0.000030
C	1.873341	-1.732635	-0.000019
C	-1.688243	-1.230461	0.000127
C	-2.786353	-2.077219	0.000218
C	-4.053443	-1.511594	0.000230
C	-4.248191	-0.136439	0.000155
C	-3.148337	0.712454	0.000063
C	-1.838315	0.167323	0.000047
C	-0.665600	1.014899	-0.000047
H	1.878941	-2.815728	-0.000023
O	-0.468311	-1.816797	0.000118
O	-0.744463	2.262923	-0.000131
O	-3.350752	2.030548	-0.000007
O	1.873552	2.393602	-0.000226
H	5.227150	-1.059808	-0.003766
H	4.464842	-2.388424	0.884587
H	4.461671	-2.394702	-0.880147
O	4.197948	1.085214	-0.000165
H	-2.633911	-3.148097	0.000276
H	-4.919097	-2.164473	0.000300
H	-5.240337	0.295843	0.000166
H	-2.478005	2.472712	-0.000065
H	0.953180	2.728985	-0.000222
H	3.974825	2.021823	-0.000285

Cartesian coordinates (XYZ format) of RVL B in the gas phase at the ωB97X-D/6-311++G level (emission state).**

C	-4.459442	-1.718093	0.000522
C	-3.125283	-1.033929	0.000196
C	-3.055263	0.375539	-0.000048
C	-1.809818	1.114614	-0.000264
C	-0.605840	0.365543	-0.000798
C	-0.686683	-1.007894	-0.000402
C	-1.931824	-1.712577	-0.000153
C	1.666822	-1.225062	-0.000160
C	2.722803	-2.111597	0.000101
C	4.016745	-1.603755	0.000349
C	4.236889	-0.228601	0.000322
C	3.176178	0.661032	0.000068
C	1.835695	0.180701	-0.000183
C	0.686276	0.991965	-0.000266
O	0.400667	-1.782754	-0.000450
O	0.789860	2.339572	0.000497
O	3.456045	1.980640	-0.000032
O	-1.869832	2.376083	-0.000040
O	-4.150682	1.112565	0.000169
H	-5.042251	-1.430650	0.879456
H	-5.042549	-1.430917	-0.878301
H	-4.339277	-2.801873	0.000670
H	2.523026	-3.175181	0.000092
H	4.860578	-2.282257	0.000579
H	5.240960	0.177325	0.000487
H	2.635438	2.490942	0.000021
H	-0.118724	2.713426	0.000101
H	-3.831710	2.037837	0.000065
H	-1.897459	-2.794830	-0.000014

Cartesian coordinates (XYZ format) of RVL B in water (IEF-PCM) at the ωB97X-D/6-311++G level (ground state).**

C	-4.391657	-1.730661	0.000075
C	-3.066275	-1.027386	0.000036
C	-3.034731	0.370198	0.000059
C	-1.824091	1.050171	0.000022
C	-0.609671	0.336298	-0.000023
C	-0.661210	-1.063469	-0.000034
C	-1.869106	-1.739104	-0.000011
C	1.691676	-1.229346	-0.000065
C	2.790635	-2.076659	-0.000069
C	4.057161	-1.511311	-0.000046
C	4.249905	-0.133479	-0.000021
C	3.149486	0.711691	-0.000022
C	1.839538	0.168083	-0.000048
C	0.666022	1.013993	-0.000061
O	0.471721	-1.814634	-0.000062
O	0.746565	2.263679	0.000080
O	3.346341	2.036323	-0.000002
O	-1.875798	2.394997	0.000016
O	-4.207580	1.058665	0.000098
H	-4.977276	-1.448417	0.878963
H	-4.977401	-1.448278	-0.878684
H	-4.259375	-2.812518	-0.000019
H	2.641983	-3.148237	-0.000082
H	4.923347	-2.162848	-0.000046
H	5.244307	0.294440	-0.000004
H	2.467643	2.469869	-0.000017
H	-0.955624	2.729637	-0.000031
H	-4.011494	2.001917	0.000141
H	-1.872183	-2.821919	-0.000020

Cartesian coordinates (XYZ format) of RVL B in water (IEF-PCM) at the ωB97X-D/6-311++G level (emission state).**

C	-4.449535	-1.713595	0.000050
C	-3.115613	-1.032107	0.000034
C	-3.037204	0.379668	0.000051
C	-1.781670	1.058006	0.000035
C	-0.575046	0.344861	0.000008
C	-0.669796	-1.048312	-0.000010
C	-1.912469	-1.720674	0.000003
C	1.688851	-1.228776	-0.000046
C	2.766871	-2.093952	-0.000068
C	4.053654	-1.553140	-0.000073
C	4.244658	-0.177545	-0.000056
C	3.145531	0.683137	-0.000034
C	1.836622	0.168250	-0.000028
C	0.671251	1.016198	0.000002
O	0.435165	-1.809677	-0.000037
O	0.736228	2.322119	0.000044
O	3.347378	2.016166	-0.000022
O	-1.785808	2.378822	0.000039
O	-4.153269	1.090016	0.000078
H	-5.035344	-1.434151	0.880553
H	-5.035388	-1.434104	-0.880410
H	-4.323961	-2.796418	0.000018
H	2.597482	-3.163100	-0.000080
H	4.911104	-2.214515	-0.000090
H	5.241158	0.247933	-0.000060
H	2.458006	2.441806	-0.000020
H	-0.800878	2.649955	-0.000001
H	-3.945097	2.035211	0.000085
H	-1.901272	-2.804302	-0.000012

Cartesian coordinates (XYZ format) of RVL B in the gas phase at the M06-HF/6-311++G level (ground state).**

C	-4.407391	-1.711441	0.000141
C	-3.060341	-1.028170	0.000041
C	-3.029801	0.366540	0.000101
C	-1.821221	1.043950	0.000011
C	-0.615571	0.323770	-0.000109
C	-0.660081	-1.072410	-0.000149
C	-1.869641	-1.746710	-0.000089
C	1.695389	-1.232000	-0.000169
C	2.797719	-2.073360	-0.000109
C	4.060689	-1.498820	0.000031
C	4.249889	-0.121560	0.000091
C	3.145429	0.718150	0.000011
C	1.844349	0.161970	-0.000119
C	0.664789	1.010630	-0.000179
O	0.476079	-1.815770	-0.000199
O	0.738359	2.244900	0.000371
O	3.330429	2.038670	-0.000009
O	-1.869781	2.387970	-0.000029
O	-4.197131	1.058469	0.000211
H	-4.970951	-1.404121	0.880471
H	-4.971151	-1.403971	-0.880009
H	-4.283361	-2.791401	0.000031
H	2.646519	-3.141510	-0.000139
H	4.927429	-2.144829	0.000081
H	5.234679	0.320071	0.000181
H	2.464779	2.489510	-0.000199
H	-0.959671	2.742740	-0.000239
H	-4.009041	2.003099	0.000231
H	-1.868091	-2.826740	-0.000129

Cartesian coordinates (XYZ format) of RVL B in the gas phase at the M06-HF/6-311++G level (emission state).**

C	-4.471591	-1.711094	-0.001045
C	-3.124168	-1.031189	-0.001195
C	-3.056187	0.372624	0.000232
C	-1.810144	1.113001	-0.001936
C	-0.588345	0.362659	0.009465
C	-0.678727	-1.018962	0.001791
C	-1.924222	-1.712693	0.001801
C	1.672276	-1.231706	0.000234
C	2.737259	-2.112199	-0.001503
C	4.022808	-1.589358	-0.002702
C	4.233358	-0.211623	-0.002269
C	3.164109	0.666796	-0.000337
C	1.836764	0.169010	0.001316
C	0.671309	0.983925	0.003329
O	0.415054	-1.783290	0.001337
O	0.784968	2.332962	0.006278
O	3.423783	1.988330	-0.000421
O	-1.851628	2.364006	-0.007376
O	-4.153826	1.106268	-0.003203
H	-5.036858	-1.412427	0.881245
H	-5.037507	-1.411552	-0.882613
H	-4.344074	-2.791029	-0.001596
H	2.542429	-3.173691	-0.001897
H	4.871149	-2.257386	-0.004106
H	5.228913	0.206915	-0.003287
H	2.605133	2.503297	0.001042
H	-0.115348	2.727230	-0.000447
H	-3.879536	2.040110	-0.004580
H	-1.888104	-2.792827	0.000236

Cartesian coordinates (XYZ format) of RVL B in water (IEF-PCM) at the M06-HF/6-311++G level (ground state).**

C	4.405590	-1.717330	0.000060
C	3.060950	-1.028070	-0.000020
C	3.029920	0.367970	0.000030
C	1.821800	1.045730	-0.000060
C	0.615510	0.325300	-0.000170
C	0.660510	-1.070930	-0.000200
C	1.870070	-1.746400	-0.000140
C	-1.695370	-1.232060	-0.000130
C	-2.797700	-2.074710	0.000020
C	-4.060770	-1.501200	0.000230
C	-4.250150	-0.121940	0.000280
C	-3.146160	0.715950	0.000100
C	-1.844350	0.161710	-0.000100
C	-0.665120	1.010300	-0.000270
O	-0.475270	-1.814250	-0.000200
O	-0.740530	2.245580	0.000390
O	-3.327520	2.041560	0.000030
O	1.866990	2.390890	-0.000120
O	4.199330	1.059650	0.000120
H	4.970300	-1.415060	-0.881340
H	4.970210	-1.415030	0.881510
H	4.274980	-2.796150	0.000070
H	-2.649320	-3.143590	0.000010
H	-4.927410	-2.147170	0.000360
H	-5.237580	0.314530	0.000410
H	-2.456910	2.486090	-0.000260
H	0.955890	2.743680	-0.000330
H	4.017360	2.006100	0.000140
H	1.870090	-2.826730	-0.000150

Cartesian coordinates (XYZ format) of RVL B in water (IEF-PCM) at the M06-HF/6-311++G level (emission state).**

C	-4.472404	-1.686439	0.000050
C	-3.119130	-1.018878	0.000037
C	-3.034532	0.390058	0.000039
C	-1.774994	1.066256	0.000039
C	-0.560324	0.318765	0.000084
C	-0.665983	-1.077401	-0.000001
C	-1.918239	-1.729515	0.000002
C	1.689910	-1.243802	-0.000056
C	2.781236	-2.095116	-0.000083
C	4.054360	-1.531061	-0.000081
C	4.234895	-0.150481	-0.000053
C	3.129187	0.694035	-0.000018
C	1.835058	0.153709	-0.000017
C	0.649728	0.983317	0.000041
O	0.444619	-1.822143	-0.000052
O	0.698415	2.301030	0.000108
O	3.302358	2.032239	-0.000040
O	-1.707418	2.359972	0.000011
O	-4.143455	1.112480	0.000024
H	-5.038707	-1.391000	0.883518
H	-5.038633	-1.391184	-0.883528
H	-4.347217	-2.766321	0.000167
H	2.625363	-3.163452	-0.000105
H	4.919236	-2.177958	-0.000108
H	5.223619	0.285095	-0.000069
H	2.418584	2.462847	-0.000080
H	-0.622882	2.584006	-0.000074
H	-3.947759	2.059869	0.000000
H	-1.920367	-2.811211	-0.000027

Supporting Code S1. Python Script for Processing and Apodization Analysis of ^{13}C NMR Spectra

```
# Install required library
!pip install nmrglue -q

import os
import zipfile
import nmrglue as ng
import numpy as np
import matplotlib.pyplot as plt

# ==========================================================================
# 1. DATA EXTRACTION
# ==========================================================================
zip_file = 'file.zip'  # Replace if your file name is different
extraction_dir = './extracted_nmr_data'

if os.path.exists(zip_file):
    print(f"Extracting {zip_file}...")
    with zipfile.ZipFile(zip_file, 'r') as zip_ref:
        zip_ref.extractall(extraction_dir)
else:
    print("Please upload the .zip file to Colab first.")

# ==========================================================================
# 2. AUTO-DETECTION OF THE 13C EXPERIMENT
# ==========================================================================
def find_13c_folder(root_directory):
    print("\nScanning for 13C experiment folder...")
    folders_13c = []

    # Recursive search for Bruker-style numbered folders
    for root, dirs, files in os.walk(root_directory):
        if 'fid' in files and 'acqus' in files:
            try:
                # Read experiment metadata
                dic, _ = ng.bruker.read(root)
                # Check observed nucleus
                nuc1 = dic.get('acqus', {}).get('NUC1', '').upper()

                if '13C' in nuc1 or 'C13' in nuc1:
                    print(f"✅ 13C experiment found at: {root}")
                    folders_13c.append(root)
            except Exception:
                continue

    return folders_13c

# ==========================================================================
# 3. PROCESSING AND PLOTTING (APODIZATION)
```

```
# ==========================================================================
def process_and_plot(data_path, ppm_range=(180, 185), lb_values=[1.0, 5.0, 10.0, 15.0]):
    print(f"\nProcessing data from: {data_path}...")
    dic, data = ng.bruker.read(data_path)
    data = ng.bruker.remove_digital_filter(dic, data)

    udic = ng.bruker.guess_udic(dic, data)
    sw_hz = udic[0]['sw']
    size = data.shape[-1]
    t = np.arange(size) / sw_hz

    fig, ax = plt.subplots(figsize=(10, 8))
    fig.patch.set_facecolor('white')

    vertical_offset = 0
    plot_scale = 1.0
    colors = ['#1f77b4', '#ff7f0e', '#2ca02c', '#d62728', '#9467bd']

    for i, lb in enumerate(lb_values):
        # Exponential apodization window
        em_window = np.exp(-np.pi * lb * t)
        apodized_fid = data * em_window

        # Zero filling (64k points for smoothness)
        fid_zf = ng.proc_base.zf_size(apodized_fid, 65536)

        # FFT and phase correction
        spec = ng.proc_base.fft(fid_zf)
        spec_phased = ng.process.proc_autophase.autops(spec, 'acme')
        spec_real = spec_phased.real

        # PPM axis
        uc_zf = ng.fileiobase.uc_from_udic(ng.bruker.guess_udic(dic, fid_zf))
        ppm_scale = uc_zf.ppm_scale()

        # Region of interest (ROI) extraction
        mask = (ppm_scale >= ppm_range[0]) & (ppm_scale <= ppm_range[1])
        ppm_roi = ppm_scale[mask]
        spec_roi = spec_real[mask]
        spec_roi = spec_roi - np.min(spec_roi)  # Manual baseline correction

        if i == 0:
            plot_scale = np.max(spec_roi)

        spec_roi_norm = spec_roi / plot_scale
        offset = i * 1.5

        color = colors[i % len(colors)]
        ax.plot(ppm_roi, spec_roi_norm + offset, label=f'LB = {lb} Hz',
                color=color, linewidth=2.5)
        ax.fill_between(ppm_roi, offset, spec_roi_norm + offset,
```

```
                  color=color, alpha=0.1)

    # ===========================================================================
    # Publication-quality styling
    # ===========================================================================
    ax.set_xlim(ppm_range[1], ppm_range[0])  # NMR convention: x-axis inverted

    ax.set_xlabel(r'Chemical Shift ($\delta$, ppm)', fontsize=16, fontfamily='serif')
    ax.set_ylabel('Relative Intensity', fontsize=20, fontfamily='serif')
    ax.set_title('Effect of Apodization on the SNR of C-9 (182.5 ppm)',
                 fontsize=20, fontfamily='serif', pad=20)

    ax.tick_params(axis='x', labelsize=14)
    ax.set_yticks([])  # Y-axis without tick labels

    ax.legend(loc='upper right', frameon=True,
              prop={'family': 'serif', 'size': 14})
    ax.grid(True, axis='x', linestyle='--', alpha=0.5)

    # Closed box frame with thicker borders
    border_width = 1.5
    for spine in ['top', 'bottom', 'left', 'right']:
        ax.spines[spine].set_visible(True)
        ax.spines[spine].set_linewidth(border_width)

    plt.tight_layout()
    plt.show()

# ===========================================================================
# 4. PIPELINE EXECUTION
# ===========================================================================
if os.path.exists(extraction_dir):
    target_folders = find_13c_folder(extraction_dir)

    if target_folders:
        # If multiple 13C folders are found, the first is processed by default
        # (typically the standard 1D spectrum; others may be DEPT experiments)
        process_and_plot(target_folders[0], ppm_range=(180, 185), lb_values=[1, 5, 10, 15])
    else:
        print("No 13C experiment was found in the extracted data.")
```
